# Ultra‐Sensitive Nanofiber‐Based Triboelectric Nanogenerator for Energy Harvesting and Self‐Powered Sensing

**DOI:** 10.1002/adma.202521626

**Published:** 2025-12-30

**Authors:** Sajib Roy, Bhaskar Dudem, Md Delowar Hussain, Vlad Stolojan, Seyedeh Sadrieh Emadian, Satheesh Krishnamurthy, Jae Sung Yun, S. Ravi P. Silva

**Affiliations:** ^1^ Advanced Technology Institute Department of Computer Science and Electronic Engineering University of Surrey Guildford Surrey UK; ^2^ Z‐PULSE Ltd. New Cambridge House Bassingbourn Road Royston Cambridgeshire UK; ^3^ Institute For Sustainability University of Surrey Guildford Surrey UK; ^4^ Surrey Ion Beam Centre University of Surrey Guildford Surrey UK; ^5^ Korea Institute of Science and Technology (KU-KIST) Graduate School of Converging Science and Technology Korea University Seoul Republic of Korea

**Keywords:** borophene, electrospinning, self‐powered sensors, triboelectric nanogenerators, wearables

## Abstract

Triboelectric nanogenerator (TENG) represents a major advancement in capability for self‐powered sensors, with its ability to convert low‐frequency mechanical movements into electricity. These devices serve at present an unmet medical and societal need in the monitoring of human activity and enhancing interactions between humans and machines, the optioned interface for setting up verifiable digital twins. Here, a novel composite nanofibrous TENG (CNF‐TENG) based on borophene@poly(vinylidene fluoride‐co‐hexafluoropropylene) (PVDF‐HFP) is constructed through electrospinning. Comprehensive materials characterization of the exfoliated nanosheets confirms crystalline sheet morphology and validates their incorporation into the fibers. The inclusion of borophene introduces a dual innovation by enhancing both the jet stressing in electrospinning and the quality of doped films. This improvement is attributed to the enhanced effective permittivity through interfacial polarization, which promotes β‐phase formation, electron‐donating capacity, surface charge trapping, and refined fiber morphology, while inducing a transition from a hydrophobic to a superhydrophobic surface state. When paired with nylon 66 nanofibers, the CNF‐TENG exhibits a remarkable sensitivity of 53.8 ± 1.2 V kPa^−1^, and a power density of 1.2 W m^−2^, representing a 13‐fold enhancement over pristine PVDF‐HFP. An array of 16 ultra‐sensitive CNF‐TENG sensors for possible use in dementia monitoring and sleep disorder mitigation is successfully demonstrated, giving various sleep patterns and physiological data sets.

## Graphical abstract

A multidimensional molecular strategy optimizes the tribonegative layer by electrospinning PVDF‐HFP for dipole alignment and crystallinity, followed by 2D borophene doping to enhance conductivity and charge trapping. Integrated with Nylon‐66 nanofibers, the composite device delivers a record sensitivity of 53.8 ± 1.2 V kPa^−^^1^ at 3 Hz, enabling ultra‐sensitive sensing for advanced wearable and biomedical applications.

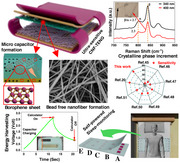

## 1 Introduction

The rapid advancement of wearable electronics and the Internet of Things (IoT) has enhanced the possibility of autonomous sensing technologies, which is critical for sustainability monitoring and looking after an ageing population. Triboelectric nanogenerators (TENGs) have emerged as a promising alternative to battery‐powered sensors, which are often constrained by limited lifespan, maintenance requirements, and environmental concerns [1, 2]. This technology is particularly advantageous due to its high output performance, light‐weight structure, high flexibility, unobtrusiveness, cost‐effectiveness, structural simplicity, and broad material compatibility [3, 4, 5, 6, 7]. Continuous efforts to improve TENG performance focus on enhancing dielectric properties, increasing surface charge density, and integrating hybrid energy harvesting mechanisms [8, 9]. In this context, electrospinning is a widely recognized technique for the production of thin films, with applications spanning various fields, including energy storage, sensing, textiles, and filtration. The advantages of electrospun nanofibrous films include high specific surface area, inherent roughness, hierarchical porous structures, and a uniformly distributed fibrous network, which can significantly enhance their surface charge density and facilitate the generation of nanofibers from viscous liquid materials [10, 11]. PVDF‐HFP is widely recognized as an effective tribonegative material due to its exceptional dielectric properties and the presence of highly electronegative fluorine groups [12]. Despite the inherent advantages of PVDF‐HFP, TENGs constructed with this pure/electrospun polymer frequently demonstrate limited triboelectric output, which may fall short of practical requirements. To mitigate this issue, the incorporation of nanofillers such as dielectric materials [13, 14, 15], and 2D materials [16, 17, 18] into the PVDF‐based polymers has been identified as a strategic necessity. Incorporation of these nanofillers enhances triboelectric performance by creating electron‐trapping sites, forming microcapacitors, increasing the relative permittivity of the base material, and promoting further crystallization.

Li et al. [19] introduced a stretchable and waterproof TENG through the electrospraying styrene‐ethylene‐butylene‐styrene (SEBS) onto a PVDF‐HFP nanofiber, which significantly enhanced the device's stretchability and waterproof capabilities, achieving a maximum contact angle of ∼140°, a maximum power density of 219.66 mWm^−2^, and a pressure sensitivity of 0.68 V kPa^−1^. Guo et al. [20] developed a pressure sensor based on a PVDF‐HFP membrane, which exhibited a sensitivity of 2.2 V kPa^−1^ within the pressure range of 0 to 2.8 kPa. Zheng et al. [21] developed a composite film of Chitosan@starch with FEP, which demonstrated a high sensitivity of 46.03 V kPa^−1^. The approach discussed above has significant potential for enhancing TENG performance, though further progress in dielectric properties, surface charge density, device stability, and power density is necessary to improve their effectiveness, sensitivity, and durability. This research emphasizes the use of tribonegative materials, particularly borophene, due to its unique molecular structure and versatile applications. Borophene's chemical and electronic properties enable strong bonding with various materials [22, 23, 24]. It is electron‐deficient, highly reactive with functional groups such as hydroxyl, amino, and carboxyl, which enhances its compatibility with polymers, metals, and ceramics. Its high surface energy facilitates robust interfacial adhesion, forming covalent interactions with polymer matrices like PVDF‐HFP. Its conductive nature enhances dielectric properties and charge density within electrospun fibers, leading to thinner, more uniform fibers with better elongation. Currently, no comprehensive studies exist on borophene@PVDF‐HFP composite nanofibers aimed at enhancing TENG performance.

In this work, a novel highly tribonegative borophene@PVDF‐HFP nanofiber mat was developed through electrospinning. The aim was to enhance the performance of the CNF‐TENG and to showcase its potential as a self‐powered pressure sensor, as well as a suitable energy source for smart wearable and portable electronic devices. By integrating the 2D borophene with PVDF‐HFP, the composite nanofibers exhibited improved dielectric constant, surface charge density, and charge‐contributing capabilities. This enhancement was achieved by promoting the formation of micro‐capacitors and incorporating a functional unit, ultimately leading to improved output performance of the proposed CNF‐TENG. Various filler concentrations (0 to 2 wt.%) of the borophene@PVDF‐HFP composite nanofibers were fabricated and optimized. In this context, an electrospun nylon 66 nanofiber mat was employed as an effective tribopositive material to increase surface charge density further and enhance overall output performance. The 1 wt.% borophene@PVDF‐HFP based CNF‐TENG led to an increase in the peak‐to‐peak open circuit voltage, short‐circuit current, and short‐circuit charge by roughly 3‐fold, 2‐fold, and 2.4‐fold, respectively, compared to pure PVDF‐HFP nanofibers. The constructed CNF‐TENG was thoroughly evaluated and tested to confirm its suitability for charging energy storage devices and powering commercially available portable or wearable electronics. The CNF‐TENG demonstrated its versatility as a self‐powered device capable of monitoring bed occupancy, toss and turn.

## 2 Results and Discussion

### 2.1 Material Characterizations, Fabrication, and Structural Design of the CNF‐TENG

**FIGURE 1:**
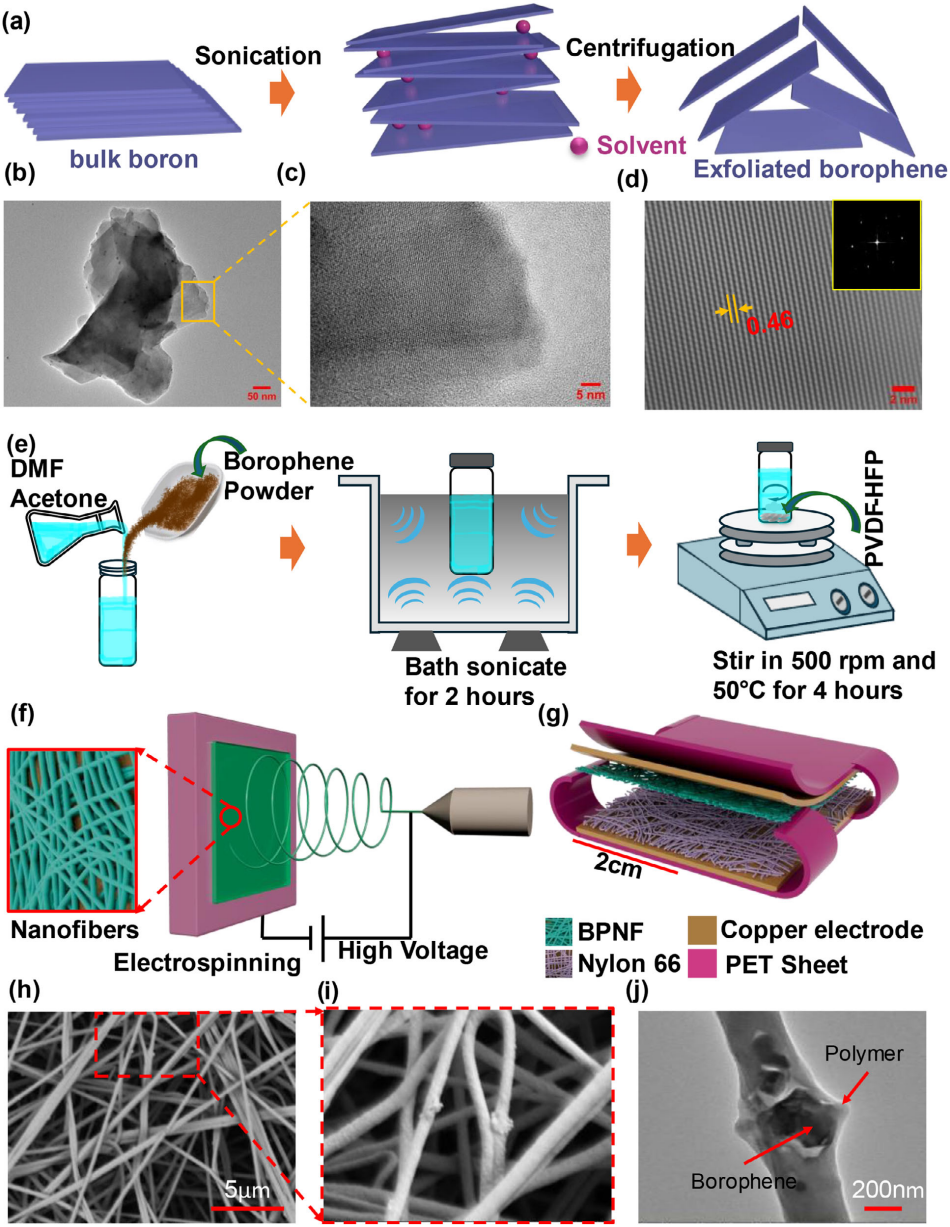
Fabrication and characterization of borophene@PVDF‐HFP composite nanofibers. Illustration of (a) Borophene synthesis process. (b,c) TEM images of exfoliated borophene sheets and the zoomed‐in region. (d) Inverse FFT of a uniform HRTEM region (inset FFT pattern). (e) Borophene@PVDF‐HFP composite solution preparation. (f) Schematic of the electrospinning process to fabricate borophene@PVDF‐HFP composite nanofiber mat. (g) A schematic design of the proposed CNF‐TENG device. (h) SEM image of the fabricated borophene@PVDF‐HFP composite nanofiber mat (1 wt.%). (i) Zoom view of borophene@PVDF‐HFP nanofiber mat. (j) TEM image showing the presence of borophene inside the borophene@PVDF‐HFP composite nanofiber mat.

The synthesis process of exfoliated borophene from bulk boron is shown in Figure 1a. Initially, the bulk boron undergoes several centrifugation steps, followed by multiple sonication steps. The detailed step‐by‐step synthesis process is described in the methods section. The photograph of the bulk boron and exfoliated borophene is shown in Figure S1a,b. The surface morphology of the bulk boron and the exfoliated borophene were carried out using Scanning Electron Microscopy (SEM) and Transmission Electron Microscopy (TEM). The SEM image of the bulk boron powder in Figure S2a had an initial particle size of around 4 µm. In contrast, the exfoliated borophene sheets are almost transparent and range in size from hundreds of nanometers to several micrometers, as shown in Figure S2b. This shows that the bulk boron was effectively exfoliated. FTIR analysis was conducted to detect the chemical bonds and functional groups present on the surface of both the bulk boron and exfoliated borophene flakes, where bulk boron and borophene flakes exhibit very similar patterns when their chemical bonds are stretched, as shown in Figure S3a. However, bulk boron's spectrum displayed sharper and stronger peaks compared to borophene's spectrum. In particular, the spectrum of borophene exhibits broader and weaker peaks compared to bulk boron, suggesting a more disordered bonding environment likely caused by sonication‐induced disruption of the atomic arrangement during synthesis. The peak in the range of 1025–1133 cm^−1^, identified as B─B stretching, is less intense and broader in borophene compared to bulk boron. The peak at 1227 cm^−1^ is associated with B─O stretching and likely originate from the presence of chemical groups containing both boron and oxygen on the surface of the borophene [25]. Following the exfoliation process, a new peak was observed at 1380 cm^−1^, which is associated with the B─O bonds of BO_3_ [26]. Raman spectroscopy is a reliable way to analyze the atomic bonding present in materials. To study the structure of borophene at a molecular level, Raman measurements were performed with the borophene placed on an Al substrate. This Al substrate was chosen because it has a blank Raman spectrum of its own, which minimizes any interference from the substrate in the analysis, and it can enhance the Raman signal from the borophene itself [27]. Raman spectra were used to reveal the crystallographic phases and vibrational response of the borophene. The spectrum of bulk boron matches β‐rhombohedral boron (Figure S3b), with bands at 274, 374, 434, 702, 792, 903, 1060, and 1152 cm^−1^, assigned to B_1u_^2^, B_1g_^2^, B_3g_^1^, A_1g_, A_g_^2^, A_g_^2^(S), B_1g_^1^, and A_g_^1^ vibration modes, respectively [28, 29]. The A_g_^1^ and A_g_^2^ modes correspond to in‐plane B–B stretching in β‐rhombohedral boron and are in the higher‐frequency range. Compared with bulk boron, the exfoliated borophene shows increased intensity in the low‐frequency region and decreased intensity at higher frequencies [29]. Moreover, the peak positions of exfoliated borophene were shifted to higher frequencies (blueshifts) compared to bulk boron. The intensity redistribution is consistent with reduced interlayer coupling and orientation/size effects in thin sheets, while the blueshift can arise from defect‐induced stiffening and the emergence of new crystallographic facets during exfoliation. In addition, the borophene spectra contain bands whose positions are consistent with reported β_12_ and χ_3_ type borophene motifs, namely the features near 434, 702, 792 cm^−1^ (β_12_‐like) and 1052, 1152 cm^−1^ (χ_3_‐like) [30]. Figure S4 shows the X‐ray Diffraction (XRD) analysis, which illustrates the crystal structures of bulk boron and exfoliated borophene flakes. The primary peaks in both materials correspond to β‐rhombohedral boron (JCPDF 00‐031‐0207), recognized as the fundamental unit of few‐layered borophene [31]. Specifically, peaks at 11.4°, 16.1°, 17.6°, 18.3°, 19.1°, 20.1°, 21.0°, 22.2°, 23.8°, 24.6°, and 26.8° correspond to the (003), (110), (104), (021), (113), (015), (006), (024), (211), and (122) planes, respectively. The crystallite size was estimated from the (104) reflection using the Scherrer relation, $D=\frac{k\lambda }{\beta \cos\theta }$ where *K*,  *λ*,  *β* and *θ* are the shape factor, X‐ray wavelength, FWHM (in radians), and the Bragg angle. The (104) peak at 2θ*θ* = 17.6° exhibits FWHM = 0.41° for bulk boron and 0.45° for the exfoliated sample, yielding *D* = 19.6 nm and 17.9 nm, respectively. The slight broadening after liquid‐phase exfoliation, therefore, indicates a modest reduction in coherent domain size, consistent with the separation of layers into thinner crystalline units. The β‐boron framework is retained after exfoliation, as evidenced by the persistence of the same set of planes, although small changes in relative peak intensities suggest preferred orientation differences in the nanosheets. Moreover, several reflections in the 16°–22° range are weaker and broader in the exfoliated material, which we attribute to defect generation and strain introduced by vigorous sonication [31, 32]. The boron's surface composition and chemical bonding were further analyzed before and after employing sonochemical exfoliation using XPS. The survey spectra with binding energies from 100 to 800 eV are illustrated in Figure S5. Apart from the B peaks, peaks attributed to C, O, and small amounts of N and F were also observed in both samples. The C, O, and minimal N, F species primarily result from surface contamination upon exposure to air. Since XPS is intrinsically surface‐sensitive, higher apparent concentrations of O and C are expected, especially for high–surface‐area flakes with many edges and defect sites [33]. It is important to note that the B and C contents of the samples changed from 73.0% to 56.6% and 10.4% to 25.7%, respectively, indicating that the surface of the borophene was contaminated by the air more than the bulk boron. The B 1s spectrum reveals two distinct peaks for bulk boron at 187.7 and 189.1 eV (Figure S6a), indicating two bonding environments for boron. The prominent peak at 187.7 eV is attributed to B─B bonds, aligning with the reported range for bulk boron (187.3–187.9 eV) [33]. Meanwhile, the peak at 189.1 eV is linked to boron suboxide (B─O), or a contribution from B─C formed during handling/solvent exposure is also possible. Deconvolution shows that approximately 76.5% of the boron signal arises from B─B bonding, implying some O/C incorporation that generates defect sites that are more reactive and thus preferentially attacked during sonochemical exfoliation, facilitating fragmentation of bulk boron. Notably, for the exfoliated sample the main B 1s component appears at 187.4 eV (Figure S6b), a slight negative shift (0.3 eV) relative to bulk. Such a modest change is consistent with preserved B─B bonding and is within the range affected by differential charging, band bending, and reference calibration to C 1s = 284.8 eV. This behavior is in line with prior reports on thin boron sheets, where the core level remains a B─B bond with a slight shift attributed to surface/edge effects [34, 35]. Figure S6c,d present the C 1s spectra, which comprise four components at approximately 282.7, 284.8, 286.3, and 288.8 eV, corresponding to C─B, C─C, C─N/C─O, and C═O, respectively. The C─B fraction in the exfoliated sample is low (∼0.03), indicating that most boron atoms remain in B─B environments after sonication, and the increased oxidized‐carbon components reflect greater surface/edge exposure of the nanosheets. Transmission electron microscopy (TEM) has been used to investigate crystallographic configurations of borophene. Drop‐cast TEM of the exfoliated product reveals thin, plate‐like nanosheets with irregular polygonal outlines and edge folds/wrinkles, confirming a 2D sheet morphology (Figure 1b). High‐resolution TEM (HRTEM) of single flakes shows continuous lattice fringes extending over tens of nanometers (Figure 1c), evidencing long‐range crystallinity. From the HRTEM, FFT of a selected region were computed, masked the principal Bragg spots, and generated an inverse FFT image that isolates the lattice contribution (Figure 1d), inset. The inverse FFT displays periodic fringes with an interplanar spacing of d = 0.46 nm, obtained from multiple line profiles indicative of the β_12_ phase of borophene [23]. Figure 1e shows a diagram illustrating the process of preparing the solution of borophene@PVDF‐HFP, where borophene powder and PVDF‐HFP pellets dissolve in the solvent to form a homogeneous solution. The composite borophene@PVDF‐HFP and nylon 66 were fabricated by using the electrospinning process. Figure 1f illustrates the fabrication process of the borophene@PVDF‐HFP composite nanofiber mat. Photographs of the electrospinning setup and the collector showing the nanofiber mat are shown in Figure S7a,b respectively. Figure S7c shows the thickness of the composite nanofiber mat is 80 microns. The developed triboelectric nanogenerator is schematically shown in Figure 1g, where the nylon 66 nanofiber mat acted as the positive triboelectric layer, while the composite borophene@PVDF‐HFP worked as the negative triboelectric layer and a highly conductive Cu tape was used for the charge‐collecting layer. Both triboelectric layers were shielded by a Polyethylene terephthalate (PET) shell (0.96 microns) using thin, flexible double‐sided tape with two sets of strips, further confirming the CNF‐TENG's cyclic contact separation depended on the applied force. Figure S8 shows the full fabrication process of the CNF‐TENG with external wiring. The PET sheets were selected because of their exceptional strength and long‐term stability, which allowed for quick separation of the layers when they come into contact. This resulted in excellent stability and high output production even at low applied force.

**FIGURE 2:**
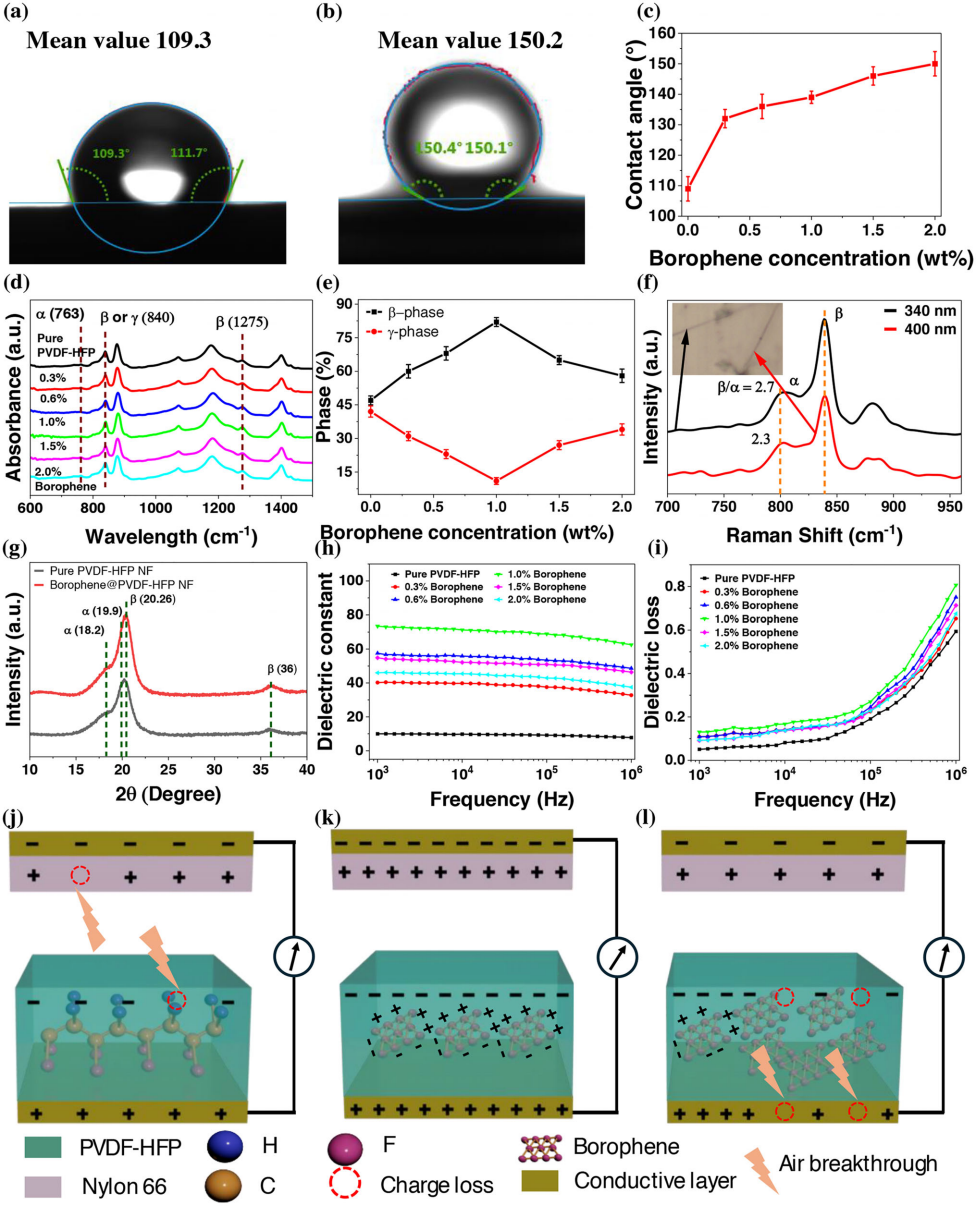
Mean water contact angle of (a) Pure PVDF‐HFP nanofiber mat (b) Borophene@PVDF‐HFP composite nanofiber for 2 wt.% borophene and (c) Increasing the mean contact angle with borophene concentration. (d) FTIR Spectrogram of pristine PVDF‐HFP nanofiber mat and borophene@PVDF‐HFP composite nanofiber mat. (e) Crystalline phase (β and γ) changes with borophene concentration. (f)Variation of β/α phases from Raman spectra of borophene@PVDF‐HFP composite nanofiber for different nanofiber diameters. (g) XRD pattern of pristine PVDF‐HFP nanofiber and borophene@PVDF‐HFP composite nanofiber. (h,i) The dielectric constant and dielectric loss of the composite nanofiber at different borophene concentrations as a function of frequency. Schematic of the formation of a micro‐capacitor into a (j) PVDF‐HFP Nanofiber, and borophene@PVDF‐HFP composite nanofiber with (k) Low and (l) High loading of borophene.

The effect of including the concentration of borophene in the polymer solution influenced the diameter and overall morphology of the nanofibers, which is observed by SEM as shown in Figure 1h,i. A significant reduction in both the number of defects (bead content) and the average fiber diameter was noted when borophene was added to the standard PVDF‐HFP solution Figure S9a–f. Typically, the beaded fibers are more amorphous in comparison to the fine fibers. In an amorphous PVDF‐HFP, the molecular chains are not arranged regularly like in crystalline PVDF‐HFP. This lack of order results in a random orientation of the dipole moments, lowering the net polarization and leading to poor triboelectric performance. Due to borophene being a good conductor, it enhances the conductivity and charge density (electrospinning jet carries a stronger overall electrical charge) of the solution. This stronger charge on the jet interacts more intensely with the electrical field during the process. The resulting stronger electrostatic stretching and stronger bending instability produce finer, more uniform fibers at fixed voltage‐flow‐distance. Adding 1 wt.% borophene increases the effective permittivity of the spinning solution via interfacial polarization; dispersed conductive flakes separated by the polymer behave as a dense microcapacitor network (explained in the latter part of this section). The higher permittivity, together with a modest rise in solution conductivity, allows the jet to sustain more surface charge at a given field, strengthening Coulombic repulsion along the jet. These conditions are known to lower the onset voltage for jet/bending instability and promote greater jet stretching, which is consistent with the observed reduction in mean fiber diameter and narrower diameter distribution at fixed voltage–flow–distance. Figure S10a shows the nanofiber diameter distribution with a histogram. The average fiber diameter of the PVDF‐HFP nanofibers decreased from 324 nm without borophene to 180 nm for containing 2% borophene (Figure S10b). Figure S11a,b shows the solution preparation followed by the electrospinning setup and a SEM image of the electrospun nylon 66 nanofibers, respectively. The thickness of the nylon 66 nanofiber was about 100 µm, as illustrated in Figure S12c. In the electrospinning process, when borophene is added to the PVDF‐HFP solution, it is anticipated that the borophene particles will align co‐axially within the nanofibers. This alignment is caused by dielectrophoretic forces resulting from the differences in dielectric or conductivity properties between the borophene and the PVDF‐HFP solution [36]. The significant shear forces produced while electrospinning are expected to play a role in this incorporation. The TEM image in Figure 1j revealed the composite nanofiber morphology, showing that the borophene nanoparticles were effectively embedded within the PVDF‐HFP polymer. The water contact angle test was carried out to determine how borophene impacts the wettability performance of electrospun PVDF‐HFP. The pure PVDF‐HFP nanofiber mat is hydrophobic with a contact angle of 109°, as shown in Figure 2a. As the concentration of borophene nanosheets increases, the contact angle of the nanofiber mat also increases significantly and reaches a mean value of 150.2° for 2 wt.% borophene, as illustrated in Figure 2b. According to Cassie‐Baxter theory, this improvement happens as a result of adding the borophene nanoparticles reduces the fiber diameter, resulting in roughening up the surface of the fiber mat [37]. Moreover, due to the presence of polar functional groups or atoms on the surfaces, borophene and PVDF‐HFP are both polar materials. Attractive forces such as dipole‐dipole interaction or Van der Waals forces can occur between their polar regions. Because of these attractive forces, borophene dispersed homogenously throughout the PVDF‐HFP when electrospun into thin fibers. This enhanced hydrophobicity, or water repulsive property, is desirable for materials that need to resist dirt and other contaminants. The contact angles of the nanofiber mats are 132°, 136°, 139°, 146°, and 150° for the borophene concentration of 0.3, 0.6, 1, 1.5, and 2 wt.%, respectively, as shown in Figure 2c and Figure S12a–d.

The relative presence of various crystalline phases (α, β, and γ) (Figure S13) in PVDF‐HFP polymers before and after adding borophene has been identified through optical characterizations, including FTIR (Fourier transform infrared spectroscopy), XRD (X‐ray diffraction), and Raman spectroscopy. The footprints of FTIR peaks of PVDF‐HFP are located at 614, 763 cm^−1^ (CF_2_ bending) and 975 cm^−1^ (CH_2_ rocking) for α‐phase, at 510 and 1171 cm^−1^ (CF_2_ rocking, CH_2_ bending), 1275 for β‐phase, and at 811, 833, and 1234 cm^−1^ for γ‐phase as shown in Figure 2d. The peak at 840 cm^−1^ can be commonly defined as the β, γ, or both phases, and 763 cm^−1^ is defined as the α‐phase. The percentage ratio of predicted electroactive β and γ‐phases (*F*_EA_) with respect to crystalline phases in any PVDF‐HFP samples can be determined as follows [38]:

(1) $$F_{EA}\left(\%\right)=\frac{I_{\beta }}{\left(\frac{K_{\beta }}{K_{\alpha }}\right)I_{\alpha }+I_{\beta }}\times 100$$

where, *I*_β_ and *I*_α_ are the absorption intensities at 840 and 763 cm^−1^, respectively; and the absorption coefficient of β and α‐phases are *K*_β_ = 7.7 × 10^4^ and *K*_α_ = 6.1 × 10^4^ respectively. The equation assumes that only the α and β‐phases are present, but in this particular region, the signal from the γ‐phase overlaps with that of the β‐phase. This overlap makes it difficult to distinguish their individual contributions. Estimating the quantities of the β and γ‐phases is possible based on the absorbance at specific wavenumbers of 1275 and 1234 cm^−1^. However, a more precise and reliable method involves finding the peak‐to‐valley height ratio between the peaks near 1275 and 1234 cm^−1^ and their closest valleys according to the equations below:

(2) $$F_{\beta }\left(\%\right)=F_{EA}\times \left(\frac{\nabla A_{\beta }}{\nabla A_{\beta }+\nabla A_{\gamma }}\right)\times 100$$

(3) $$F_{\gamma }\left(\%\right)=F_{EA}\times \left(\frac{\nabla A_{\gamma }}{\nabla A_{\beta }+\nabla A_{\gamma }}\right)\times 100$$

where, ∇*A*_β_ represents the difference in absorbance between the peak at approximately 1275 cm^−1^ and the closest valley at around 1260 cm^−1^. Meanwhile, ∇*A*_γ_ represents the difference in absorbance between the peak at approximately 1234 cm^−1^ and the closest valley at around 1225 cm^−1^. The relative fraction of β and γ‐phases for all samples was calculated based on Equation (1), and the individual amount of β and γ‐phases were calculated using Equations (2 and 3). As the concentration of borophene nanosheets increases from 0 to 1 wt.%, the β‐phase increases from 47% to 82% (Figure 2e). Meanwhile, for the borophene concentration of 1.5 and 2 wt.%, it decreases slightly to 65% and 58%, respectively. As expected, β increases with borophene loading while γ decreases (anti‐correlated trends), which is consistent with β‐stabilization at the expense of γ (Figure 2e). Incorporating borophene promotes the β phase via multiple synergistic mechanisms such as stronger local electrospinning fields that produce thinner fibers and align dipoles (C‐F alignment), heterogeneous nucleation at polymer–nanosheet interfaces, where polar/oxidized surface sites template and stabilize all‐trans segments, and interfacial polarization from a micro‐capacitor network, which raises effective permittivity and deepens charge traps, supporting polar ordering. These effects collectively increase β‐phase crystallinity and the sustainable surface charge that governs the triboelectric output. Although the increased β‐phase indicates enhanced intrinsic piezoelectric activity, the device architecture used here (Figure 1g) does not form the electrode–polymer–electrode configuration required for efficient piezoelectric charge extraction. Because one side of the active layer is contacted by nylon 66 rather than a conductive electrode, the piezoelectric displacement current experiences a very high impedance pathway and is therefore strongly attenuated, making its contribution negligible compared with the triboelectric effects. The optimum at intermediate loading is consistent with pre‐percolation enhancement followed by post‐percolation aggregation/leakage that reduces both β content and performance. These findings consistently show that incorporating borophene nanosheets strengthens the development of the β‐phase in borophene@PVDF‐HFP nanofiber composites, enhancing the material's piezoelectric potential while simultaneously improving dielectric properties. This suggests that electrospinning combined with 2D materials like borophene offers a powerful approach to producing electroactive β‐phase PVDF‐HFP. We performed a Raman analysis to support the results obtained from the FTIR spectrum. Raman spectra of pristine PVDF‐HFP and borophene@PVDF‐HFP were obtained using a green 532 nm laser to understand more about the relationship between the crystalline phase quantity and nanofiber diameter. Two distinct bands are visible at around 794 and 839 cm^−1^, corresponding to the α‐phase and the β‐phase, respectively. The Raman spectra of pristine PVDF‐HFP nanofiber for different fiber diameters ranging from 250 nm to 540 nm are shown in Figure S14. This indicates that the peak ratio of β‐phase and α‐phase changes significantly from 1.8 to 2.5 with the changes in nanofiber diameter from 250 to 540 nm. Compared to pure PVDF‐HFP, the crystalline component ratio of 1% borophene@PVDF‐HFP, as shown in Figure 2f, increases from 2.1 (300 nm) to 2.7 (340 nm). The XRD patterns of pure PVDF‐HFP nanofiber and Borophene@PVDF‐HFP nanofiber (1 wt.% borophene) depicted in Figure 2g. The diffraction peaks identified at 18.2°, 19.9°, 26.3°, and 35.8° correspond to the (020), (110), (021), and (200) crystal planes of the α‐phase. Furthermore, the peaks observed at 20.26° and 36.0° are representative of the β‐phase, while the major peak at 20.04° and two moderate peaks at 18.5° and 39.0° relate to the (110/101), (020), and (211) crystal planes of the γ‐phase. With the addition of borophene to the electrospinning solution, the intensity of the peaks at 20.26° and 36 associated with the β‐phase, gradually increased, whereas the peak at 18.2° linked to the α‐phase exhibited a significant decrease. A peak shift for pure PVDF‐HFP is also visible from 20.26° toward 19.9°, associated with the α‐phase. This finding is consistent with the results obtained from the FTIR and Raman analysis.

The dielectric constant is a key parameter for boosting TENG output. Thus, increasing the permittivity of the tribolayer is expected to enhance device performance. Figure 2h shows the frequency‐dependent dielectric properties of PVDF‐HFP nanofibers as a function of borophene loading (0–2.0 wt.%) over 10^3^ − 10^6^ Hz. The dielectric constant of the films were measured as described in the method section. Incorporating borophene into the composite nanofiber produces a pronounced rise in dielectric constant. The dielectric constant gradually reduces as frequency increases, because the impact of space charge polarization diminishes [39]. At 1 kHz, the relative permittivity increases from the pure PVDF‐HFP nanofiber baseline (∼10) to a substantially larger value (∼75) for 1 wt.% borophene@PVDF‐HFP. Increasing the amount of borophene fillers led to a decrease in the dielectric constant, indicating that the filler reached the percolation threshold in the borophene@PVDF‐HFP composite. Furthermore, the loss tangent rises with increasing filler concentration, as shown in Figure 2i, confirming that the filler creates a microcapacitor network and conductive pathway. The frequency roll‐off for the dielectric constant and the loss upturn at high frequencies are consistent with the finite response time of interfacial charges and the onset of leakage at elevated loading. The composition dependence follows a percolation form,

(4) $$\varepsilon_{n}\propto \varepsilon_{p}{\left(\varphi_{T}-\varphi_{L}\right)}^{-c}\;for\;\varphi_{L}<\varphi_{T}$$

where *φ*_T_ is the critical mass fraction of the percolation threshold, *φ*_L_ is the mass fraction of the filler materials, c is the critical exponential, *ε*_n_ and *ε*_p_ are the electrical permittivity of the nanocomposite and dielectric permittivity of the insulating polymer. In the borophene@PVDF‐HFP nanofiber composite, the percolation threshold (*φ*_T_) occurs at a borophene concentration of 1 wt.%. Because *C* = *ε*_r_ *ε*_0_ *A*/*d* and *σ* = *ε*_r_ *ε*_0_Δ*V*/*d*, the measured increase in relative permittivity directly elevates the device capacitance and achievable surface charge density, explaining the concomitant gains in triboelectric performance at the optimal loading. When the filler concentration approaches the percolation threshold (*φ*_T_), the composite's dielectric constant can be described using the microscopic dipole and microcapacitor network models. In the absence of borophene, polarization charges tend to accumulate near the electrodes [40]. The quantity of induced charges present in pristine PVDF‐HFP nanofibers during the electrospinning process correlates with the amount of the piezoelectric phase, as illustrated in Figure 2j. The dielectric film's surface charge density significantly determines the TENG's performance. As per Paschen's law, the air breakdown limit governs the maximum achievable surface charge density. Enhancing the dielectric permittivity, such as incorporating a conductive intermediate layer within the dielectric material, is a key and effective method of increasing this air breakdown limit. A microcapacitor model is established when filler materials interact closely, resulting in interfacial polarization between the fillers and the polymer matrix. As the concentration of borophene increases, the distribution of microcapacitors also rises, leading to a significant enhancement of the limit of air breakdown and surface charge density. Upon the application of an external electric field, the microscopic dipole network generated through the formation of microscopic dipoles causes electric charges to accumulate at the interface between the polymer and the filler. Figure 2k displays a uniform distribution of borophene, with each particle operating as an individual capacitor structure. As the concentration of borophene increases alongside the filler fraction, the filler particles maintain a uniform dispersion. The charges present on the internal surface of the nanofiber composite facilitate greater interfacial polarization by introducing additional microcapacitors, which in turn augments the induced charge on the surface of the nanofibers. However, if the concentration of borophene surpasses a specific threshold, referred to as the percolation threshold point, the filler particles begin to aggregate, as shown in Figure 2l. At this stage, the clustering of multiple borophene particles creates a conductive pathway, potentially leading to a tunnelling effect if the particles approach each other closely enough. As a result, the microcapacitor arrangement is disrupted, causing a reduction in interfacial polarization and an electrical breakdown occurring between the dielectric material and the charge‐collecting layer. Notably, the total surface charge of the composite nanofiber reduces, leading to a significant reduction in the dielectric layer's surface charge density and the TENG's electrical output.

### 2.2 Working Principle and Simulation of the Proposed TENG

TENG converts mechanical energy into electricity using triboelectrification or contact electrification and electrostatic induction. Contact electrification is a common phenomenon that happens when two opposite‐charged materials come into contact and separate as shown in Figure S15a–d. Initially, the friction layers are separated, resulting in a neutral state with no potential difference. When borophene@PVDF‐HFP nanofiber mat contacts nylon 66 nanofiber mat under external pressure, electrons are redistributed due to differences in electron affinity. This leads to negative charges accumulating on the borophene@PVDF‐HFP surface and positive charges on the nylon 66 surface. Although the total charge remains constant, a potential difference develops as the layers move apart, inducing opposite charges on the electrodes through electrostatic induction, leading to electron flow between the nanofiber mats, and causing an instantaneous current flow through the external load (positive peak of Figure S15f. The charge flow occurs rapidly, reaching equilibrium when maximum separation is achieved, at which point the electrostatic induction stops. When the layers move closer again, increased contact area causes charges to accumulate anew, creating a potential difference that drives current in the opposite direction (negative peak of Figure S15f. Finally, when the two triboelectric layers completely come into contact, the system returns to its initial state, and no current flows through the load. Figure S15f illustrates the current flow for a complete cycle of contact and separation. In the contact separation mode TENG, there is no transfer or flow of charge under open‐circuit conditions. Therefore, the open‐circuit (OC) voltage *V_OC_* can be defined as follows [41]:

(5) $$V_{OC}=\frac{\sigma x\left(t\right)}{\varepsilon_{0}}$$

Here, *x*(*t*) is the separation distance between two nanofiber mat layers, *σ* represents the charge density of the nanofiber surface, and *ε*_0_ denotes the air permittivity.

The short‐circuit charge and short‐circuit current depend on the surface charge density of the tribomaterials, effective contact area (*S*), the separation distance, and the thickness of the dielectric nanofiber mat. The formulas for transferred charges, *Q_SC_* and short‐circuit current, *I_SC_* can be expressed as follows [41]:

(6) $$Q_{SC}=\frac{S\sigma x\left(t\right)}{d_{0}+x\left(t\right)}$$

(7) $$I_{SC}=\frac{dQ_{SC}}{dt}=\frac{S\sigma v\left(t\right)}{{\left(d_{0}+x\left(t\right)\right)}^{2}}$$

Here, *v*(*t*) represents the velocity of the relative movement of the dielectric layers. In order to comprehend the operational mechanisms and perform a quantitative assessment of the electric potential distribution within the TENG, a simulation was conducted utilizing COMSOL Multiphysics through the finite element method (FEM) (Methods Section). This simulation was specifically tailored for the proposed borophene@PVDF‐HFP composite‐based TENG device. The thickness of the borophene@PVDF‐HFP nanofiber mat was established at 80 µm, and the nylon 66 nanofiber mat was 100 µm. Figure S16a,b illustrates the simulated electrical potential distribution in the TENG concerning the separation distance between the borophene@PVDF‐HFP and nylon 66 nanofiber surfaces. Nonetheless, the correlation between the output voltage and the gap between the layers (spanning from 0.5 to 10 mm) displays nonlinear characteristics, as shown in Figure S16c. The nonlinear deviation arises from the nonlinear behavior of capacitance with gap. As the gap increases, the capacitance reduces nonlinearly, and so does the induced electric potential.

### 2.3 Electrical Performances of the TENG

**FIGURE 3:**
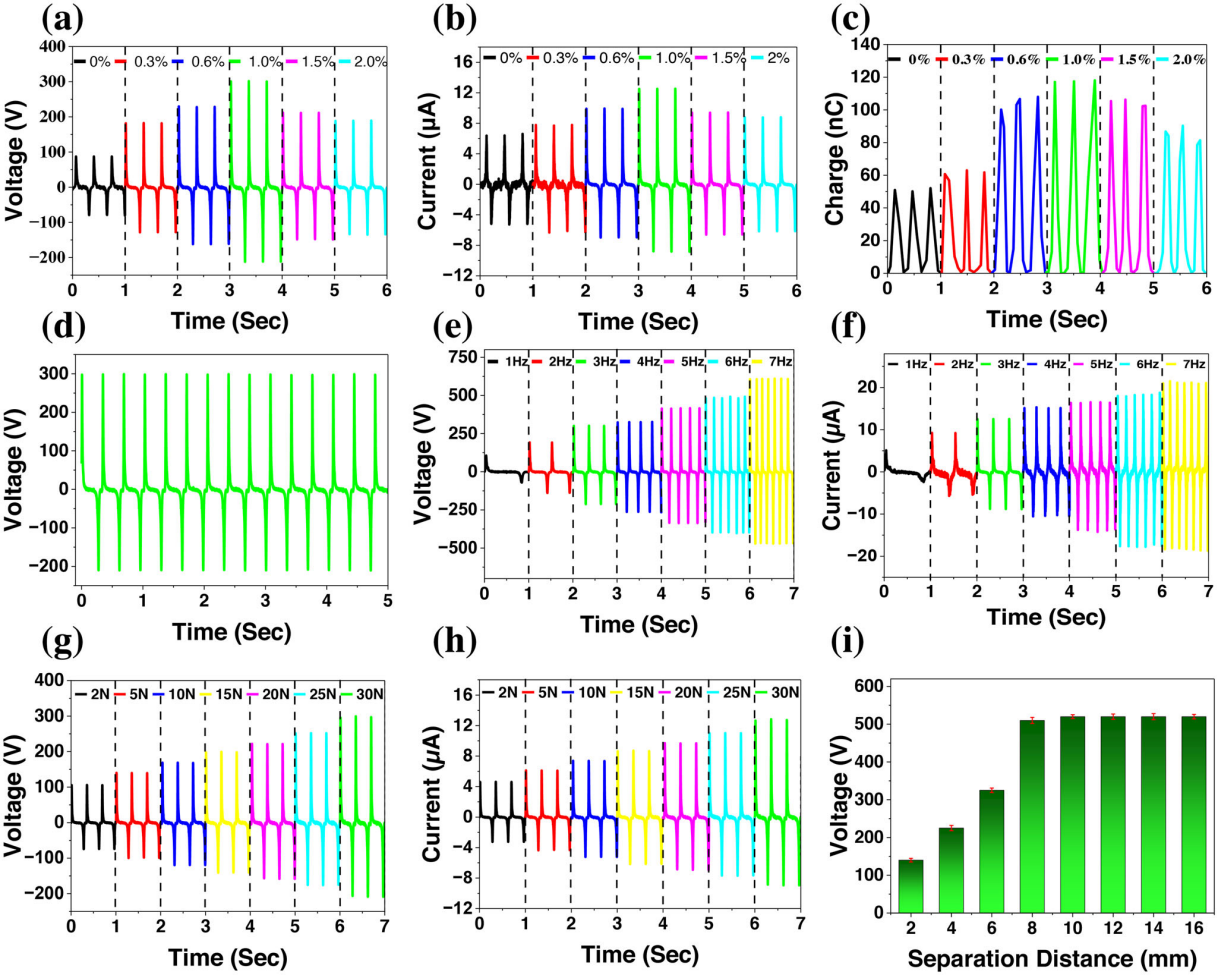
Investigation of the electrical properties of the CNF‐TENG based on different parameters. Changes in (a) open circuit voltage. (b) Short circuit current and (c) transferred charge with different loading of borophene from 0‐2 wt.% at an applied force of 30 N and operating frequency of 3 Hz. (d) Open circuit output voltage for a borophene concentration of 1 wt.%. (e) Peak to peak voltage open‐circuit and (f) short‐circuit current of the optimized CNF‐TENG at a constant applied force of 30 N and various excitation frequencies ranging from 1–7 Hz. (g) Peak to peak open circuit voltage and (h) short circuit current of the optimized CNF‐TENG at a constant operational frequency of 3 Hz and various applied forces ranging from 2 to 30 N. (i) Open circuit voltage curves, indicating different distances between the tribomaterials.

The operational efficiency of the CNF‐TENG device's electrical output was evaluated based on different scenarios, such as peak‐to‐peak open‐circuit voltage, short‐circuit current, and transferred charge. This assessment was conducted at a frequency of 3 Hz and 30 ± 5 % relative humidity, utilizing an experimental setup specially designed for TENG as the photograph presented in Figure S17. Table S1 exhibits the geometry, the testing conditions used for the electrical characterization, and the electrical features of the TENG. To improve the electrical output performance of the device, borophene@PVDF‐HFP nanofibers were used as the tribonegative layer, while nylon 66 nanofibers served as the tribopositive layer measuring 4 × 4 cm^2^. An extensive study was performed to optimize the electrical output efficiency of the TENG by testing various concentrations of borophene. The effects of different concentrations of borophene mixed with the PVDF‐HFP composite on the output performance were also examined, analyzing the electrical output across a range of concentrations from 0 to 2 wt.%. The open circuit voltage, short circuit current, and transfer charge of the CNF‐TENG device for different borophene concentrations in the nanofibers mat were illustrated in Figure 3a–c, respectively. The employed frequency and applied force to obtain these results are 3 Hz and 30 N, respectively. As the concentration of borophene increased within the PVDF‐HFP composite nanofiber from 0 to 1 wt.%, the peak‐to‐peak open‐circuit voltage, short‐circuit current, and transfer charge increased from 160 to 510 V, 11.6 to 21.3 µA, and 50 to 120 nC, respectively. The 1 wt.% borophene@PVDF‐HFP based CNF‐TENG led to an increase in the open circuit voltage, short‐circuit current, and short‐circuit charge by roughly 3‐fold, 2‐fold, and 2.4‐fold, respectively, compared to pure PVDF‐HFP nanofibers. The enhanced triboelectric performance of the CNF‐TENG device due to the integration of borophene into the PVDF‐HFP composite because of the improved crystalline phase (β‐phase) and dielectric properties of the composite materials. Prior to reaching the percolation threshold (up to 1 wt.%) the capacitance of the dielectric layer increases with the concentration of borophene in the polymer layer. It is associated with the enhancement of micro‐capacitors and small dipoles in the composite polymer layer, resulting in a maximized charge density in the TENG. The enhanced charge accumulations due to the formation of micro‐capacitors improve charge‐tapping abilities and polarization until it hits the percolation threshold of the filler material. As the concentration of borophene increases from 1 to 2 wt.%, the performance of the device noticeably decreased. In particular, the output peak‐to‐peak open‐circuit voltage, short‐circuit current, and transferred charge dropped to 320 V, 14.8 µA, and 62 nC. This finding indicates that an excess of borophene, although it only resulted in a minor reduction in resistivity due to the extra filler content surpassing the percolation threshold, formed a conductive pathway through direct interaction with the filler. The voltage signal displayed noticeable asymmetry, likely due to adhesion‐induced impulsive separation [42]. After contact, the interaction between the triboelectric surfaces created a “sticking effect” that changed the input motion profile, resulting in sudden separation of the TENG layers and increasing the peak voltage of the relevant half‐cycle. This phenomenon may be affected by a mix of dielectric parameters, the gap between dielectric layers, and operational circumstances [43].

The composite nanofiber mat made of borophene@PVDF‐HFP with a 1 wt.% borophene filler demonstrated the highest electrical performance. As illustrated in Figure 3d, the output peak‐to‐peak open‐circuit voltage attained levels of 510 V. Figure S18a,b shows the charge and current density at different concentrations, indicating that the device performs optimally at 1 wt.% borophene filler. The CNF‐TENG was further studied using the composite nanofibers with an optimized 1 wt.% borophene concentration because of its exceptional output performance. The study investigated how the input excitation frequency affects the CNF‐TENG's performance. The findings indicate that the CNF‐TENG's output voltage and current increase proportionately with changes in input frequencies, as shown in Figure 3e,f. The open‐circuit voltage and short‐circuit current increase from 175 to 1080 V and 7.5 to 40.2 µA, respectively, as the frequency changes from 1 to 7 Hz. Although the performance of the CNF‐TENG improves with increasing frequency, 3 Hz is selected for subsequent experiments due to its relevance to the wearable band. 3 Hz resides within the upper spectrum of human motions addressed in this study (sleep micro‐movement). In Figure 3g,h, the output performance is illustrated when subject to changes in applied forces from 2 to 30 N at a frequency of 3 Hz. The experiments reveal a linear growth in open‐circuit voltage and short‐circuit current from 175 to 510 V and 7.8 to 21.3 µA, respectively, with the incremental applied force from 2 to 30 N. Figure 3i depicts the output peak‐to‐peak open‐circuit voltage of the proposed CNF‐TENG as a function of varying separation distances between the dielectric materials, which range from 2 to 16 mm. The results indicate that the output voltage rises from 140 to 510 V as the distance increases from 2 to 8 mm. Beyond this point, the open‐circuit voltage stabilizes, with only a slight increase, peaking at 520 V, when the separation distance reaches 10 mm or greater. The saturation occurs because there is a maximum limit to the quantity of surface charge density that a material can hold, dictated by dielectric constant and electrical breakdown. Once this limit is reached, further separation does not generate an additional charge or voltage. Surface charge saturation occurs when the charge density exceeds the dielectric breakdown threshold of the material.

**FIGURE 4:**
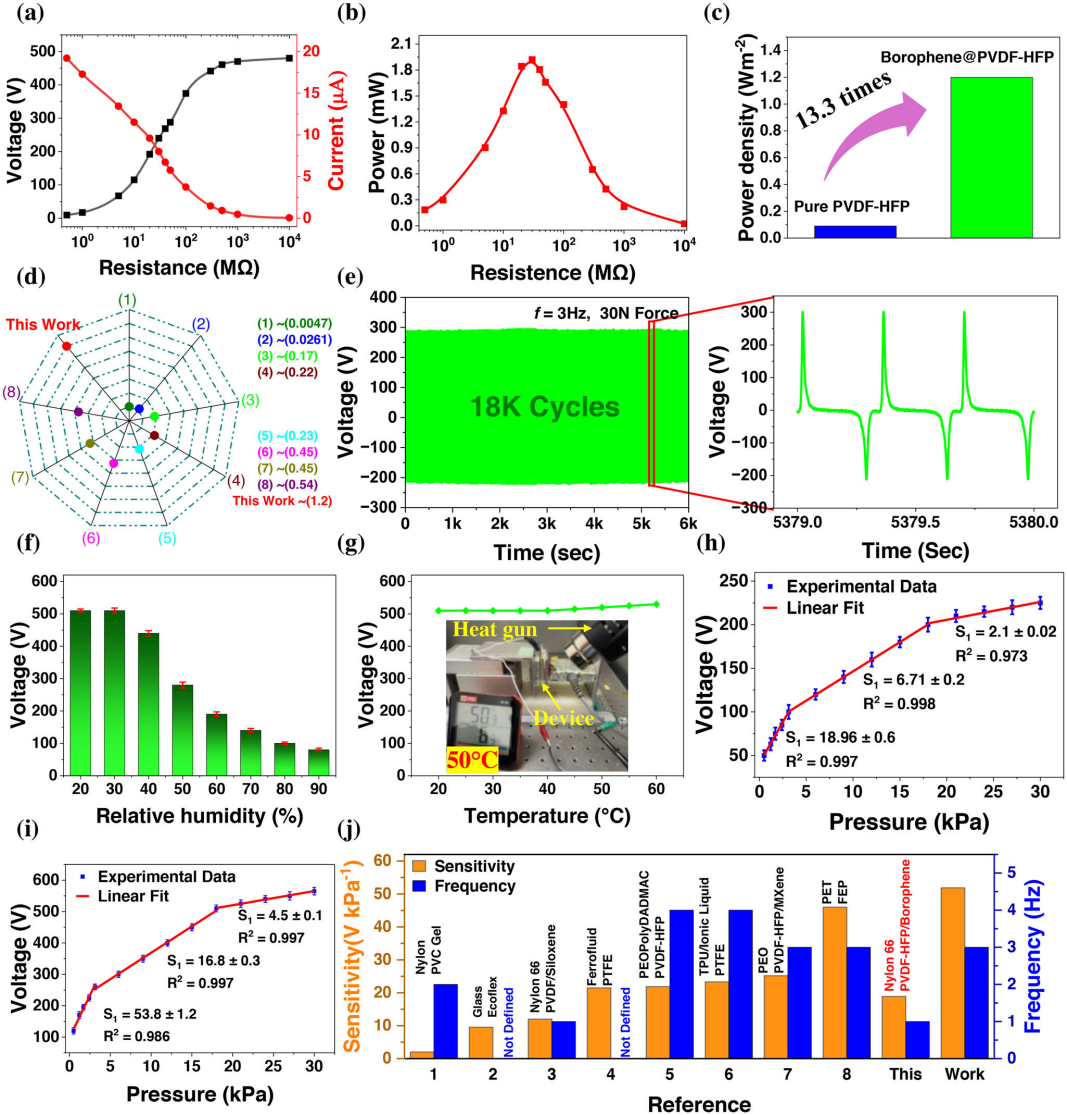
The output performance of the CNF‐TENG operating at an applied force of 30 N and an operating frequency of 3 Hz. (a) Changes in voltage and current across different external load resistances, (b) output power with different load resistance, representing the impedance matching at a resistance value of 30 MΩ. (c) Comparison of power density of pure PVDF‐HFP nanofiber and optimized CNF‐TENG. (d) Power density comparison with recently published PVDF‐HFP composite materials based on the TENG literature: (1) [45], (2) [46], (3) [47], (4) [19], (5) [16], (6) [48], (7) [49], (8) [18]. (e) Durability test of the CNF‐TENG for 18,000 cycles at a frequency of 3 Hz and applied force of 30 N. (f) Changes in output voltage with changes in humidity from 20% to 90%. (g) Changes in output voltage with changes in temperature from 20°C to 60°C. (h) Linear fit of pressure versus voltage waveform to get the sensitivity of the CNF‐TENG at (i) 1 Hz and (j) 3 Hz operational frequencies. (j) Sensitivity comparison of the CNF‐TENG with recently reported TENG‐based self‐powered pressure sensors 1 [50], 2 [51], 3 [52], 4 [53], 5 [54], 6 [55], 7 [56], 8 [21].

To evaluate the practical and commercial potential of the CNF‐TENG as an energy source or self‐powered sensor, it is essential to determine its capability to generate maximum power output for a specific load. To achieve this, a series of experiments were conducted by connecting the CNF‐TENG to different resistors as external loads (*R*_L_) and measuring the voltage across the resistors. This data was then used to calculate the adequate electrical output power as well as the power density of the device. Figure 4a depicts that the instantaneous load voltage (*V*_L_) across the resistor increased in accordance with Ohm's law as the load resistance varied from 0.5 to 10^4^ MΩ. Conversely, the current exhibited an inverse relationship with the voltage. Ultimately, the voltage reached its maximum under open‐circuit conditions as the load resistance attained very high values, whereas the current reached its maximum under short‐circuit conditions when the load resistance was low. Figure 4b and Figure S19a illustrates how the output peak power and power density vary with external load resistance. These figures clearly indicate that, as the resistance increases, both the output power and power density of the device rise initially, reaching a peak before beginning to decrease. The peak‐to‐peak power and power density was calculated utilizing the following formula, [44] $W=V_{L}^{2}/R_{L}$ and $W=V_{L}^{2}/SR_{L}$, respectively where *S* represents the effective contact area of the device. The maximum peak‐to‐peak output power of the CNF‐ TENG reaches around 1.92 mW and power density of 1.2 Wm^−2^ at an optimum load resistance of 30 MΩ and a load voltage of 240 V, for an applied force and frequency of 30 N and 3 Hz, respectively. Figure S19b shows the waveform of the voltage at 30 MΩ load resistance. The optimized borophene@PVDF‐HFP composite TENG demonstrates a significant improvement compared to the TENG that utilizes only pure PVDF‐HFP nanofibers, achieving an above 13‐fold increase in output power density, as illustrated in Figure 4c. This enhancement is attributed to incorporating the borophene nanofiller into the PVDF‐HFP matrix, which serves as a negative triboelectric layer.

Figure 4d and Table S2 [16, 18, 19, 46, 47, 48, 49] present a comparison of the output power density performance of the proposed CNF‐TENG with previously reported TENGs made from composite PVDF‐HFP materials. Additionally, a comprehensive comparison was carried out as shown in Table S3 with well‐established tribopositive materials, such as nylon, PET and other positive materials [57, 58, 59, 60, 61, 62, 63]. This comparison focused on key metrics, including voltage output, power density, and stability. The composite developed in this study demonstrated superior performance across these parameters, highlighting its enhanced capabilities. To further assess performance, we analyzed the durability of the CNF‐TENG devices illustrated in Figure 4e. We performed tests to evaluate their stability and long‐term functionality by measuring the output voltage after around 18,000 continuous cycles at a frequency of 3 Hz, applying a force of 30 N, while keeping the relative humidity at 30 ± 5 %. The findings from this research demonstrate that the CNF‐TENG maintains a consistent and stable voltage output over extended use, with no notable degradation detected. Enhancements in both chemical and physical modifications to the surface could improve the resilience of the composite nanofiber mat. The tests carried out affirmed the stability and durability of the constructed devices, confirming that the CNF‐TENG is suitable for extended applications.

We carried out an investigation on how humidity and temperature impact the CNF‐TENG. The setup for humidity‐controlled testing is depicted in Figure S20a. The experimental findings presented in Figure 4f demonstrate that the CNF‐TENG can provide stable performance up to 40% humidity, with a minor decline in efficiency at elevated levels. Nevertheless, the CNF‐TENG continues to produce over 280 V even at 50% humidity, making it appropriate for portable wearable devices and sensors. The significant decrease in performance at 90% humidity is likely attributable to moisture affecting the material's surface, which creates a conductive pathway for charge dissipation. The decline of open‐circuit voltage with humidity follows the known dielectric response of nanostructured polymers under moisture [64]. Capillary condensation increases the water volume fraction within surface/nanopore features, which raises ε′ and leakage conductivity via interfacial polarization and enhances *ε″*, thus *σ*_ac_(*ω*) = *ε*_0_ *ω* *ε* *′′*, accelerating tribocharge dissipation. Water films also screen surface potential and promote neutralization at separation through capillary bridges. Figure S20b illustrates the device's stability under conditions of 50% humidity (at 3 Hz and a force of 30 N), showing no considerable degradation over 18,000 cycles while maintaining an output of 280 V. Furthermore, we evaluated how temperature affects the CNF‐TENG performance by introducing hot air into the testing chamber, as shown in Figure S21a. The outcomes in Figure 4g indicate that the CNF‐TENG performance remains consistent within the temperature range of 20°C–60°C. Figure S21b depicts the stability of the device's voltage waveform at 50°C, with no noticeable deterioration over 18,000 cycles while sustaining an output voltage of 515 V. From this analysis, we deduce that the CNF‐TENG can function reliably under various humidity and temperature conditions, demonstrating its suitability for wearable applications.

To emphasize the capability of the CNF‐TENG as an instantaneous pressure sensor, we performed an investigation under different pressure conditions at a relative humidity of 30 ± 5%. Our analysis included assessments at frequencies of 1 and 3 Hz. Figure S22a illustrates the voltage output of the CNF‐TENG in response to various instantaneous pressures at a frequency of 1 Hz. The findings reveal that the electrical output voltage consistently increases with rising pressure until it reaches saturation at 225 V when the pressure applied is 30 kPa. This saturation occurs because applying 30 kPa of pressure optimizes the frictional contact area within the internal structure of the nanofiber mat, leading to the highest triboelectric surface charge generation in the CNF‐TENG. To verify that the voltage plateau above ∼30 kPa (Figure 4h–i) originates from limited tribocharge, we quantified the surface charge as a function of pressure. As shown in Figure S23, charge density rises steeply at low pressures and saturates near 30 kPa, mirroring the behavior of output voltage. This confirms that the high‐pressure regime is charge‐limited rather than instrument‐limited. The trend is consistent with breakdown/screening in air and the approach of real contact area to its geometric limit. A higher applied pressure results in a larger interaction area, which is a crucial element. The output voltage of the CNF‐TENG is greatly affected by pressure; increased pressures produce higher voltage outputs. The relationship between applied pressure and voltage is almost linear (R^2^ = 0.997), demonstrating a high sensitivity of 18.9 ± 0.6 V kPa^−1^ for pressures up to 3.125 kPa as shown in Figure 4h. After 3.125 to 18 kPa, and 18 to 30 kPa, the sensitivity drops to 6.7 ± 0.2 and 2.1 ± 0.02 V kPa^−1^, respectively, yet the relationship maintains near linearity. After 30 kPa, the output voltage achieves saturation. In conclusion, the device exhibits good sensitivity across the 1–30 kPa pressure spectrum. Figure S24a–d further depict the CNF‐TENG's output voltage under varying instantaneous pressures ranging from 1 to 30 KPa and highlight the maximum voltage output at a frequency of 1 Hz.

An additional examination of the electromechanical properties was performed to explore the pressure‐sensing capabilities of the CNF‐TENG at a frequency of 3 Hz. Figure S22b demonstrates the CNF‐TENG's output voltage under different instantaneous pressure levels. The findings reveal that within the pressure range of 1 to 18 kPa, the output voltage experiences a substantial increase, rising from an initial measurement of 180 to 510 V. The functionality of the CNF‐TENG is significantly affected by the applied pressure, as elevated pressure conditions result in a higher output voltage. This improvement in electrical output performance under varying pressures is due to the increased effective interaction area between the two nanofiber layers of the tribomaterials when greater pressure is exerted. At lower pressures, the textured surface morphology of the nanofiber mat limits full interaction between the borophene@PVDF‐HFP and nylon 66 nanofibers. As a result, only a small portion of the borophene@PVDF‐HFP interfaces with the nylon 66 nanofiber mat, leading to minimal triboelectric charge generation and, therefore, low electrical output. When additional pressure is applied to the device, it deforms to a greater extent, which increases the contact area between the borophene@PVDF‐HFP and nylon 66 nanofibers mat. This expanded contact area facilitates a higher generation of charges, resulting in an elevated voltage output. Figure S25a–d illustrates the peak‐to‐peak voltage waveform under varying instantaneous applied pressures ranging from 1 to 30 kPa at a frequency of 3 Hz. Figure 4i presents the correlation between output voltage and its corresponding standard deviation as functions of different instantaneous pressures at a frequency of 3 Hz. The results indicate a voltage–pressure relationship with two clearly distinct regions. Up to pressures of 3.125 kPa, the voltage–pressure correlation is almost linear (R^2^ = 0.986), showing a sensitivity of 53.8 ± 1.2 V kPa^−1^. In the interval between 3.125 to 18 kPa and 18 to 30 kPa, the pressure sensitivity decreases to 16.8 ± 0.3 and 4.5 ± 0.1 V kPa^−1^, yet the relationship between generated voltage and pressure remains highly linear. Beyond a pressure of 30 kPa, the CNF‐ENG reaches a saturation limit. Compared to previously reported literature [21, 50, 51, 52, 53, 54, 55, 56], this CNF‐TENG demonstrates superior pressure sensitivity (Figure 4j, Table S4), underscoring its potential for practical sensing applications.

### 2.4 Application and Demonstration

The system diagram in Figure S26 illustrates a CNF‐TENG designed to capture mechanical energy and transform it into usable electric power for portable and wearable electronic devices. This technology utilizes biomechanical energy from various human activities and converts it into electrical power. Subsequently, a full‐wave rectifier IC (DF04) is employed to convert the produced energy into direct current (DC), which is then stored in a capacitor or battery. The stored electrical energy can be used as a green energy source to power different electronic devices, such as a stopwatch, hygrometer, calculator, and wearable electronics for continuous monitoring of human movement, thereby establishing an environmentally friendly and self‐sustaining system.

**FIGURE 5:**
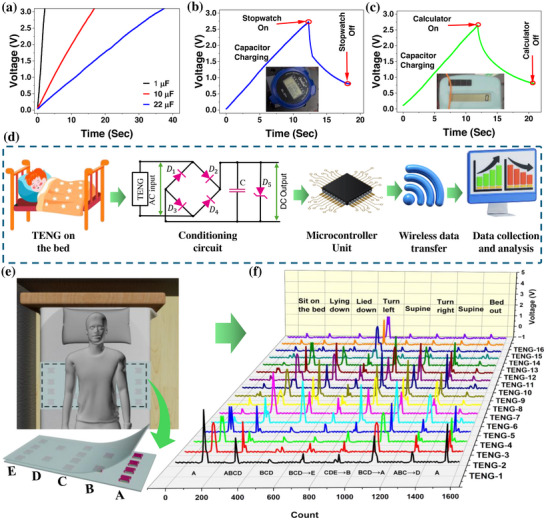
(a) Voltage waveform of charging different‐sized capacitors up to 3 V. Voltage waveform of charging and discharging a 10 µF capacitor while the CNF‐TENG is working as an energy harvester to charge a (b) stopwatch and (c) a calculator. (d) Schematic diagram of the CNF‐TENG based smart sleep activity monitoring system as a self‐powered pressure sensor. (e) Position of the sensor array on the bed with the five divided parts. (f) Response curves of the 16 sensors' data based on different sleep activities and sensor positions.

Energy harvesting performance of the CNF‐TENG have been evaluated by measuring the time it takes for charging different capacitors up to a steady voltage of 3 V (operating voltage of most IoT‐based low‐powered sensors and traditional CMOS) under the same input conditions (applied force and frequency of 30 N and 3 Hz, respectively). The results showed that capacitors with different capacitance values took varying times to reach a voltage of 3 V. Specifically, three different capacitors, including 1 µF, 10 µF, and 22 µF were charged for 1.6, 16, and 37 s, respectively, to get the 3 V level as Figure 5a. Furthermore, the CNF‐TENG was used to charge various commercial portable electronics, representing its potential as a renewable energy source. At first, a stopwatch is powered by the voltage from a capacitor, which is charged using the proposed CNF‐TENG over a certain period. By gently tapping the CNF‐TENG for 12 s, a 10 µF capacitor can be charged to 2.7 V, which is sufficient to run a commercial stopwatch for approximately 6 s. Figure 5b illustrated the capacitor charging discharging graph while powering the stopwatch. The inset at the bottom shows the stopwatch in its active state during the experiment. Similarly, tapping the CNF‐TENG for 12 s charges the same 10 µF capacitor to 2.7 V, enabling it to power a calculator for approximately 9 s, as depicted in Figure 5c. The inset at the bottom shows the calculator in its active state during the experiment. Additionally, the CNF‐TENG can continuously run low‐power electronic devices with gentle hand‐tapping. After a few seconds of hand tapping, the voltage at the capacitor reached the start voltage of the electronic devices, and continuous powering them is shown in Movies S1 and S2. The device also powered 100 series‐connected red LEDs, highlighting its potential for low‐power electronics, as shown in Movie S3. These findings demonstrate that the CNF‐TENG can generate adequate power to sustain the continuous operation of wearable electronics over an extended period.

Further demonstrations of the sensor's structural design and material characteristics enable it to track body movements, suggesting possible uses for non‐invasive, real‐time sleep monitoring. Sleep monitoring has gained significant attention due to the critical role of sleep quality in overall health and well‐being. Traditional sleep monitoring systems, however, often rely on external power sources, such as batteries, which are bulky, require frequent recharging, and wearing these devices can limit user comfort and potentially affect the quality of sleep [65]. TENGs have emerged as a groundbreaking solution to these challenges, offering a self‐powered, lightweight, and flexible platform for monitoring postural changes during sleep [66]. To track body movements in real‐time while sleeping, we developed an intelligent platform that fits underneath the bed sheet, measuring 60 cm × 30 cm. This platform consists of an array of 16 flexible CNF‐TENG sensors. The sleep‐monitoring array is presented as a standard benchmark to demonstrate that the materials advances translate to stable, self‐powered sensing under realistic, low‐pressure conditions. Initially, the CNF‐TENG sensors were placed on top of a cotton textile, and the external wiring was completed (Figure S27a). Subsequently, the sensors were covered with an additional layer of cotton textile and sewn together using a sewing machine to enhance their robustness (Figure S27b). During sleep, the real‐time data was collected using a multi‐channel microcontroller (Arduino) through a signal conditioning circuit and transferred wirelessly to a web browser Figure 5d. The signal conditioning circuit consists of a full‐wave bridge rectifier (DF04) that converts the AC input signal into DC. It includes a capacitor (100 pF) to store the signal and a 5‐volt Zener diode (1N5231B) to prevent the high voltage from damaging the microcontroller unit. Channels are read sequentially by the ADC of the Arduino and transferred the data wirelessly (WiFi) to the web browser. The 10‐bit ADC linearly mapped 0–1023 counts, which is equal to 0–5 V. In post‐processing recorded counts were converted to volts, so all plots are shown on a 0–5 V scale. Any excursions above 5 V are clipped by the Zener, ensuring safe acquisition. The surface of the array has been divided into five sections labelled A to E, as illustrated at the bottom of Figure 5e. Sensors 1 to 4 are located under section A, sensors 5 and 6 are located under section B, sensors 8 to 10 are located under section C, sensors 11 and 12 are located under section D, and sensors 13 to 16 are located under section E, as illustrated in Figure S27c. It is essential to consider the movements that occur during sleep, especially when shifting positions. The scenario of sleep patterns divided into six categories including sitting on the bed, lying down, supine, turning left, turning right, and bed out. In accordance with the typical behavior of TENG, the proposed CNF‐TENG sensor generated two distinct peaks, one upon the applied pressure and another upon its release. A rectifier integrated into the conditioning circuit ensures that both the positive and negative peaks are converted to two positive signals, where one corresponding to the applied pressure and the other to the released pressure (Figure S28). When a participant sits at the edge of the bed, only the sensors in part A register the applied pressure, resulting in the first positive signal (Figure 5f). Due to the high sensitivity of these sensors, adjacent sensors also displayed minor peaks due to movements of the bed. When the patient lies down, pressure was released from the sensors in section A, creating the second positive signal upon releasing the pressure. At the same time, the sensors in sections B, C, and D are pressed, indicating a supine position as depicted in Figure S29a. As the person turns to the left, they shift from section D back to section A. In this case, section D recorded a signal that indicates the release of pressure, as depicted in Figure S29b. Returning to the supine position involves the release of pressure from section A while pressure was applied to the sensors in sections B, C, and D, as depicted in Figure S29c. Similarly, during the left turn, the participant applies pressure to sections C, D, and E while releasing it from section B as depicted in Figure S29d. The voltage data collected from all sensors, reflecting the various movements, has been summarized in Figure 5f. A heat map of the real‐time body movement has been shown in Movie S4.

## 3 Conclusion

In conclusion, a superhydrophobic CNF‐TENG was successfully fabricated using electrospun borophene@PVDF‐HFP as the tribonegative layer and nylon 66 nanofibers as the tribopositive layer. Incorporating 2D borophene enhances the effective permittivity through interfacial polarization and promotes β‐phase formation from 47% to 82%, thereby increasing the sustainable surface charge and TENG output. The optimized device achieved a peak‐to‐peak open‐circuit voltage of 510 V and a maximum power density of 1.2 Wm^−^^2^ at a 30 MΩ load. Notably, as an energy harvester, the CNF‐TENG can supply power to low‐power electronics like a stopwatch and a calculator, even with gentle hand tapping under practical operating conditions. It also functioned as a self‐powered pressure sensor with ultrahigh sensitivity (53.8 V kPa^−1^ at 3 Hz) and enabled real‐time sleep monitoring. This study highlights borophene's multifunctionality and presents a scalable pathway for advanced TENGs in wearable and biomedical applications. Future work should focus on optimizing borophene's exfoliation and functionalization to improve structural stability, interfacial compatibility, and dielectric properties for medical monitoring applications, including heart and respiration rate tracking.

## 4 Methods

### 4.1 Materials

Polyvinylidene fluoride‐co‐hexafluoropropylene (PVDF‐HFP) pellets with a molecular weight of 455,000 Mw, nylon 66 pellets (molecular weight of 226.14), dimethylformamide (DMF) (solvent), acetone (co‐solvent), formic acid (solvent), and Boron with a purity of 95% were purchased from Sigma Aldrich, USA.

### 4.2 Borophene Synthesis

Borophene, a new group of 2D materials, has recently captured the attention of researchers due to its impressive versatility. They are potentially used in a broad spectrum of applications, such as flexible electronics, energy storage devices, energy harvesting technologies, etc. Borophene nanoparticles were synthesized using the well‐known sonochemical approach, 3 g of boron powder were dispersed in 300 mL of ethanol within a beaker. Following a 1‐h probe sonication treatment, the solution was equally distributed across 6 individual 50 mL centrifuge tubes. Each centrifuge tube containing the distributed solution was subjected to an ultrasonic bath and ultrasonicated for 4 h. The water was replaced every hour to ensure consistent temperature control during the ultrasonic bath process. After that, the solution was centrifuged for 5 min at 3000 rpm, and the bulk boron was separated from underneath by pouring the solution into different centrifuge tubes. Each new centrifuge tube containing the decanted solution underwent a subsequent centrifugation step at 8500 rpm for 30 min. After the centrifugation step, the desired components (supernatant) were separated from the solvent, and the supernatant was carefully collected and dried in a vacuum oven at 50°C overnight. This drying step ensures the removal of any residual solvent, leaving behind the isolated and concentrated product. Starting with 3 grams of boron and following the described procedure, around 100 mg of borophene powder was obtained.

### 4.3 Solution Preparation for Electrospinning

At first, pure PVDF‐HFP pellets with a concentration of 20 wt.% were dissolved in a mixed solvent of DMF and acetone with a 7:3 ratio. The solution was stirred continuously using a magnetic stirrer for 4 h at 50°C temperature and 500 rpm rotation to form a homogeneous solution. Subsequently, to produce a borophene@PVDF‐HFP composite solution, borophene powder containing different concentrations (0.3, 0.6, 1.0, 1.5, and 2.0 wt.% of PVDF‐HFP) were bath sonicated into DMF and acetone solvents with a 7:3 ratio for 30 min to disperse the borophene into the solvent. Later, PVDF‐HFP pellets with a concentration of 20% were added to the solution and stirred for 4 h at 50°C temperature and 500 rpm rotation to form a homogeneous solution. At last, nylon 6, 6 pellets (molecular weight of 226.14) containing 15 wt.% were dissolved in formic acid. To form a homogeneous solution, it was stirred continuously using a magnetic stirrer overnight at 70°C temperature and 350 rpm rotation.

### 4.4 Nanofiber Mat Fabrication

All the electrospun nanofiber mats were produced using a conventional single‐needle electrospinning setup. The fabrication process of the borophene@PVDF‐HFP composite nanofiber mat is depicted in Figure 1c. Photographs of the electrospinning setup and the collector showing the nanofiber mat are shown in Figure S7a,b, respectively. The polymer solutions were loaded into a syringe (20 mL) with a stainless‐steel needle spinneret (diameter d = 0.686 mm) gauge 19, which was placed in an automatic syringe pump (Details) in a horizontal direction to a flat collector. Silicone release paper was coated on top of the collector to collect the fiber mat. The metal needle was connected to a positive DC high‐voltage power supply to charge the solution, and the collector was connected to the ground. The DC voltage was optimized to 20 KV, and the tip‐to‐collector distance was adjusted to 15 cm for all the samples. The electrospinning process was carried out for 40 min by pumping the solution at a set flow rate of 1.5 mL h^−1^. The relative humidity and the temperature of the electrospinning chamber were around 30 ± 3% and 23 ± 1°C, respectively. For nylon 6,6, a silicone release paper was placed on top of the collector to collect the fiber mat. The applied DC voltage was 22 KV, and the tip‐to‐collector distance was adjusted to 10 cm. The electrospinning process was carried out for 1.5 h by pumping the solution at a flow rate of 0.4 mL h^−1^. The relative humidity and the temperature of the electrospinning chamber were around 30 ± 3% and 20 ± 1°C, respectively. After electrospinning, the mats (pristine PVDF‐HFP, borophene@PVD‐HFP, and nylon 6,6) were left to dry completely at 50°C temperature to a conventional oven overnight. This drying step allows any remaining solvent trapped within the fibers to evaporate before further analysis.

### 4.5 CNF‐TENG Device Fabrications

Contact separation mode CNF‐TENG devices were fabricated using the composite borophene@PVDF‐HFP (60 µm thickness) and nylon 66 nanofiber mat (100 µm thickness) as opposite triboelectric materials. Both nanofiber mats were carefully peeled from the silicone release paper and cut into 4 × 4 cm^2^ identical squares. Conductive copper tape as an electrode was then attached to the opposite sides (the side not coming into contact) of both tribomaterials. A commercially available PET sheet (95 µm thickness), which worked as a substrate, was attached to the electrodes using very thin and flexible double‐sided tape. Finally, the PET sheet was folded so that the tribomaterials faced each other with a proper gap, and external wiring was developed for electrical performance measurements (Figure S8).

### 4.6 Simulation Approach

In the AC/DC modules, a stationary electrostatic analysis evaluates the performance of the CNF‐TENG. The geometric model of the CNF‐TENG used in these simulations corresponds to the experimental configuration, which is the primary focus of this analysis. All edges of the simulation model are grounded. Electrospun nylon 66 and PVDF‐HFP serve as the positive and negative dielectrics, respectively, while copper functions as the conductive layer, with the remaining spaces filled with air, all sourced from the COMSOL Multiphysics materials library. A parametric sweep simulates the movement of contact separation. Once the parametric sweep is complete, the dataset for the open circuit voltage is generated in COMSOL and then plotted using Origin. The specific structural parameters used in the simulation are detailed in Table S1.

### 4.7 Characterizations

Scanning Electron Microscopy (SEM) was carried out using ThermoFisher Scientific Apreo SEM to examine the surface morphology of the samples in detail. Before examining the samples under the SEM, bulk boron and all the electrospun mats were sputter‐coated with a very thin layer of gold (about 6 nm) using a sputter coater (Quorum 150 V ES plus). As the borophene itself is conductive, it wasn't coated with gold. A software program called ImageJ 2.0 was used to measure the diameters of the electrospun fibers and analyze how the fiber size varied across a particular sample and with different borophene concentrations. In order to understand the polar crystalline phase contents, the Fourier Transform Infrared (FTIR) spectra were analyzed using a Varian Cary 660 FTIR spectrometer in the range of 400 to 4000 cm^−1^. Raman spectra of the samples were captured using a Horiba Xplora Plus MULTILINE Confocal Raman microscope. The microscope used 532 nm excitation (green laser), and it had a focus spot with a diameter of about 0.8 µm, employing a 100× long working distance microscope objective. To validate the composition of the crystalline structure of the samples, X‐ray diffraction (XRD) was also utilized in the range of 2θ = 10° to 70°. X‐ray Photoelectron Spectroscopy (XPS) was performed using a Theta Probe with parallel angle‐resolved XPS capability for quantitative analysis of chemical bonding states and elemental composition. Transmission electron microscopy (TEM), carried out using a ThermoFisher Scientific FEI Talos F200i, confirmed the presence of borophene nanosheets inside the PVDF‐HFP nanofibers. A Kruss Drop Shape Analyzer 25 was used to conduct the wettability test of the electrospun nanofiber mats. Dielectric characterization was performed on free‐standing PVDF‐HFP films. Film thickness was measured first, after which 12 mm diameter disks were punched and assembled in Swagelok‐type cells using stainless‐steel electrodes as current collectors. Potentiostatic electrochemical impedance spectroscopy was recorded using a Gamry Interface 1000 over the frequency range of 1 kHz–1 MHz. The complex permittivity across this frequency range was then obtained from the impedance data using Equations (8) and (9).

(8)

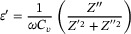



(9)

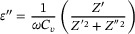

where, $C_{v}=\frac{\varepsilon_{0}S}{d}$ is the vacuum capacitance, *d* is the thickness of the nanofiber film, *S* is the area of the film, *ω* is the angular frequency, *ε*_0_ is the permittivity of the free space, and *Z*′ and *Z*′′ are real and imaginary impedance of the capacitor. The ratio of complex and real permittivity then determines the dielectric loss of the material. A digital oscilloscope (Tektronix 4 series) and a low‐noise current preamplifier (model SR570) were used to measure the open‐circuit voltage and short‐circuit current, while the transferred charge was measured using a Keithley 6514 electrometer. The contact separation was carried out using a special setup, which included a linear motor and a force sensor as shown in Figure S17.

## Conflicts of Interest

The authors declare no conflicts of interest.

## Supporting information




**Supporting File 1**: adma71953‐sup‐0001‐SuppMat.docx

**Supporting File 2**: adma71953‐sup‐0002‐MovieS1.mp4

**Supporting File 3**: adma71953‐sup‐0003‐MovieS2.mp4

**Supporting File 4**: adma71953‐sup‐0004‐MovieS3.mp4

**Supporting File 5**: adma71953‐sup‐0005‐MovieS4.mp4

## Data Availability

The data that support the findings of this study are available in the supplementary material of this article.
