# Supplementary material for: Ultra‐Sensitive Nanofiber‐Based Triboelectric Nanogenerator for Energy Harvesting and Self‐Powered Sensing
Source: Adv Mater. 2025 Dec 30;38(10):e21626. doi: 10.1002/adma.202521626 (PMC12910543; doi:10.1002/adma.202521626)
Supplement: Supplementary file 1 — Supporting File 1: adma71953‐sup‐0001‐SuppMat.docx [file ADMA-38-e21626-s001.docx]

**Supporting Information**

**Ultra-sensitive nanofiber-based triboelectric nanogenerator for energy harvesting and self-powered sensing**

*Sajib Roy, Bhaskar Dudem, Md Delowar Hussain, Vlad Stolojan, Seyedeh Sadrieh Emadian, Satheesh Krishnamurthy, Jae Sung Yun, S Ravi P Silva**


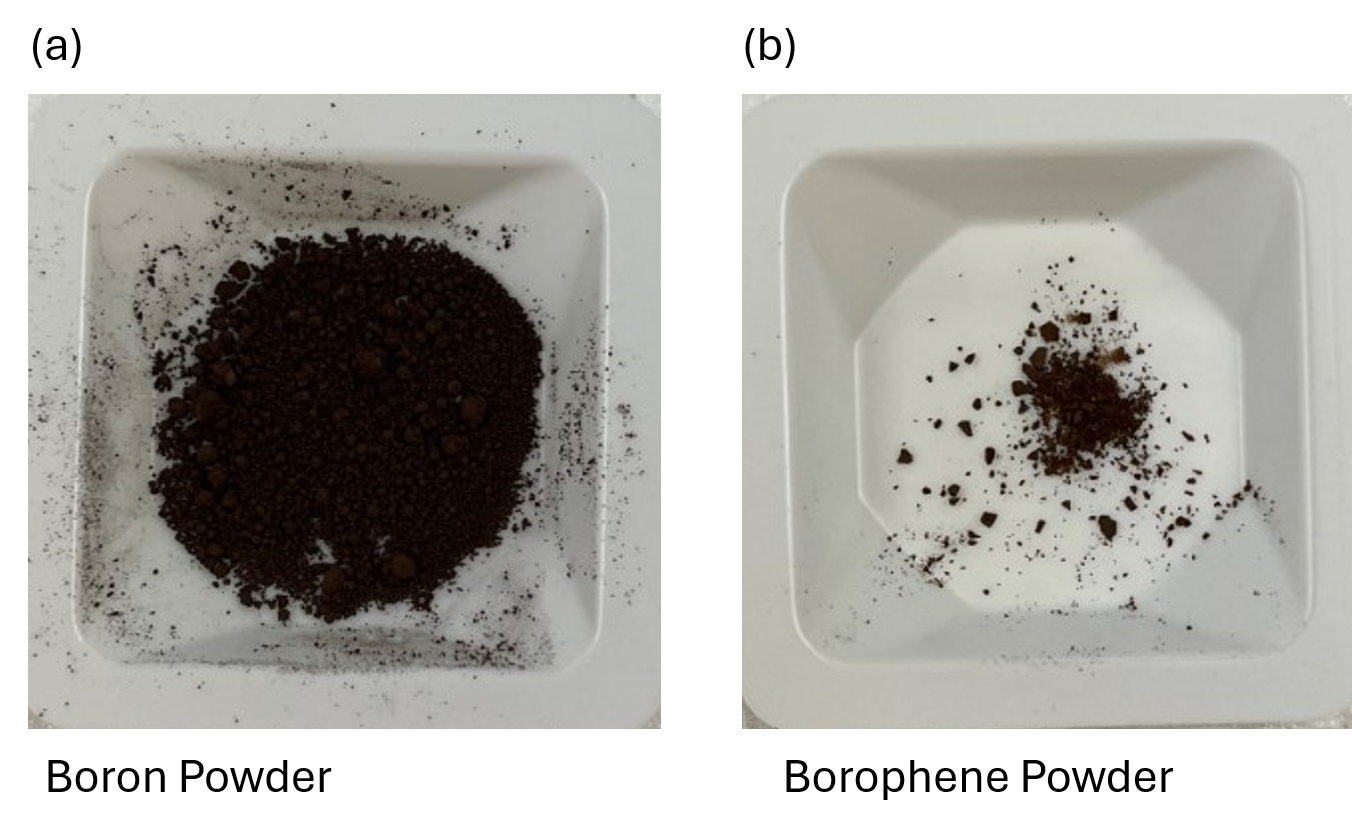


**Figure S1.** Photograph of (a) Bulk boron and (b) Borophene powder.


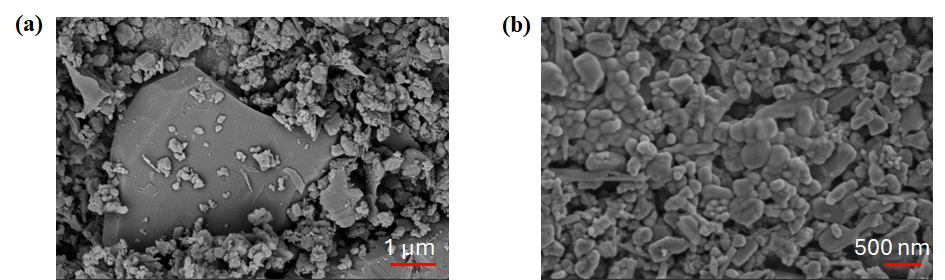


**Figure S2.** Sem images of (a) Bulk boron and (b) Exfoliated borophene.

**
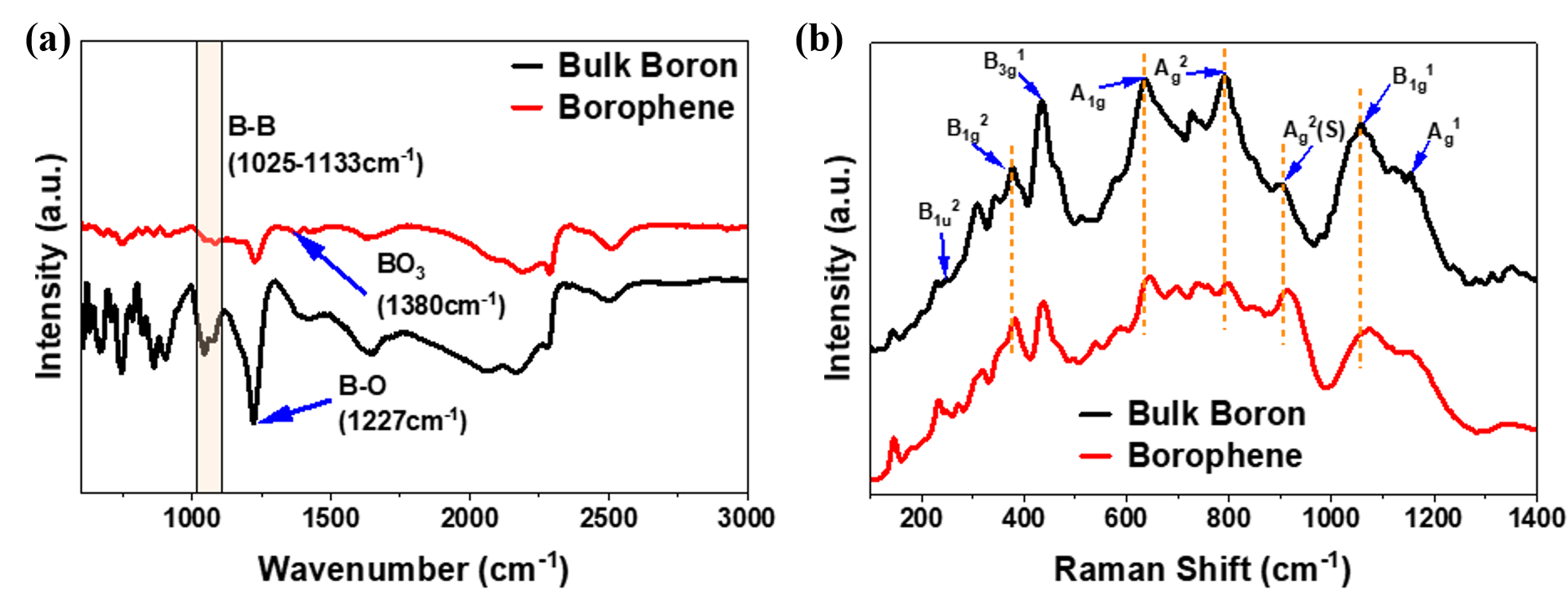
**

**Figure S3.** (a) FTIR and (b) Raman spectra of bulk boron and exfoliated borophene.

**Figure S4**. X-ray diffraction (XRD) of bulk boron and exfoliated borophene.

**Figure S5.** X-ray photoelectron spectroscopy (XPS) survey spectrum of bulk boron and exfoliated borophene.

**Figure S6.** B1s region of (a) Bulk boron and (b) Exfoliated borophene. C1s region of (c) Bulk boron and (d) Exfoliated borophene.


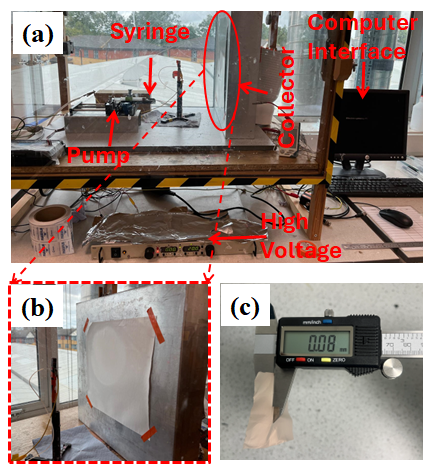


**Figure S7.** Photograph showing (a) Full electrospinning setup, (b) Silicone release paper coated collector, and (c) Thickness of the borophene@PVDF nanofiber mat.


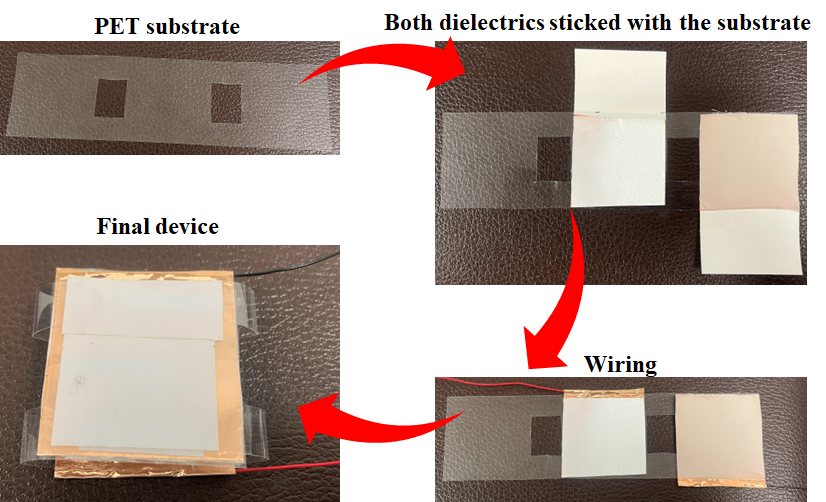


**Figure S8.** Photograph of the CNF-TENG device fabrication process


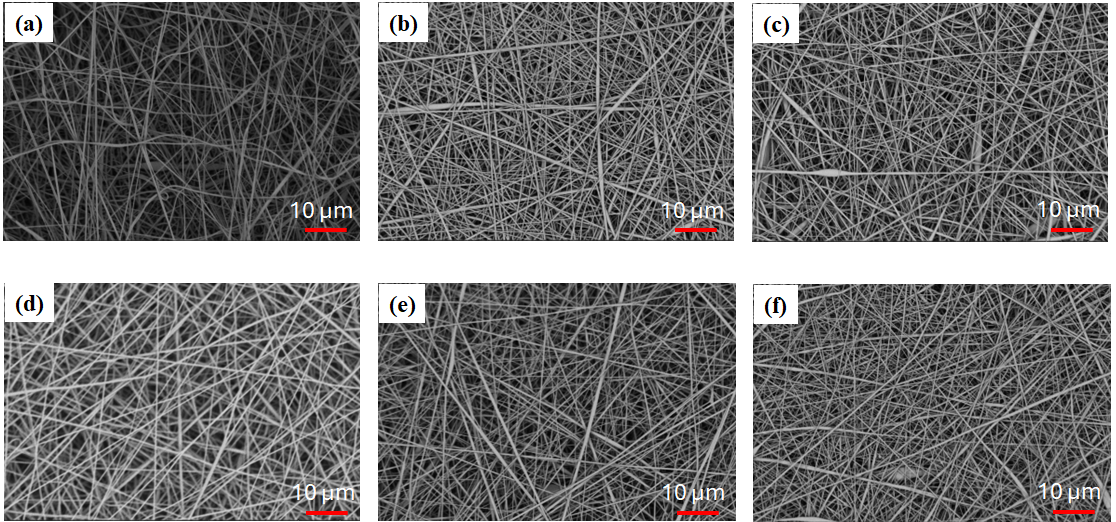


**Figure S9**. SEM images of PVDF-HFP with (a) 0%. (b) 0.3%. (c) 0.6%. (d) 1%. (e) 1.5% and (f) 2% borophene.

**Figure S10.** (a) Histogram showing the nanofiber diameter distribution. (b) The changes in mean nanofiber diameter with borophene concentration.


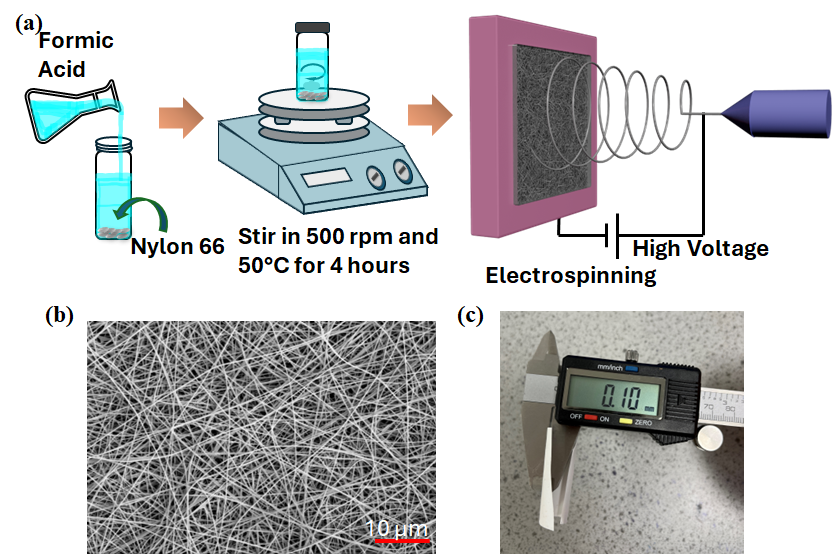


**Figure S11.** (a) Solution preparation and electrospinning of nylon 66. (b) SEM image of electrospun nylon 66. (c)Photograph showing the thickness of the electrospun nylon 66 nanofiber mat.


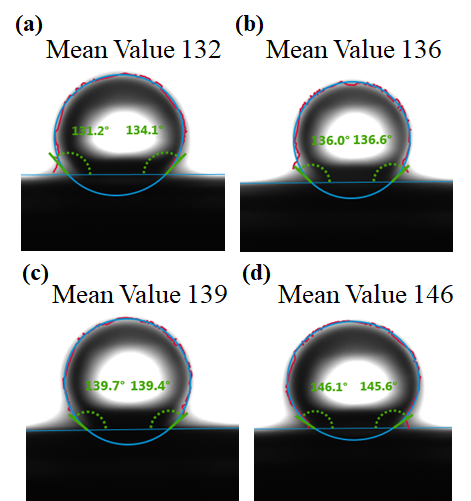


**Figure S12.** Changes of mean water contact angle of borophene@PVDF-HFP composite nanofibers for different borophene concentration of (a) 0.3 (b) 0.6 (c) 1.0 and (d) 1.5 wt%.


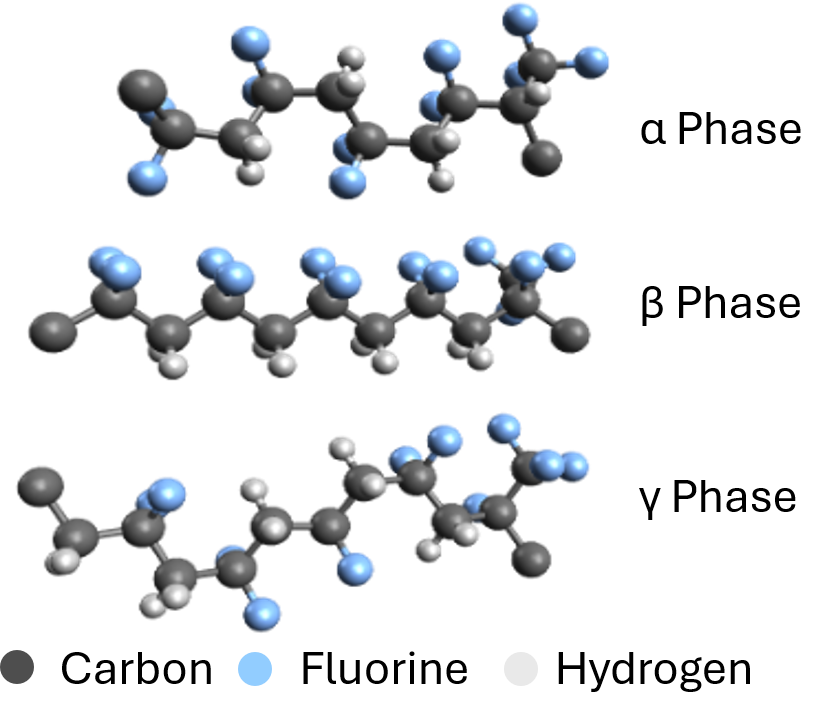


**Figure S13.** Three different crystalline phase structures of PVDF-HFP.

**Figure S14.** Variation of β/α phases from Raman spectra of pure PVDF-HFP for different nanofiber diameters.


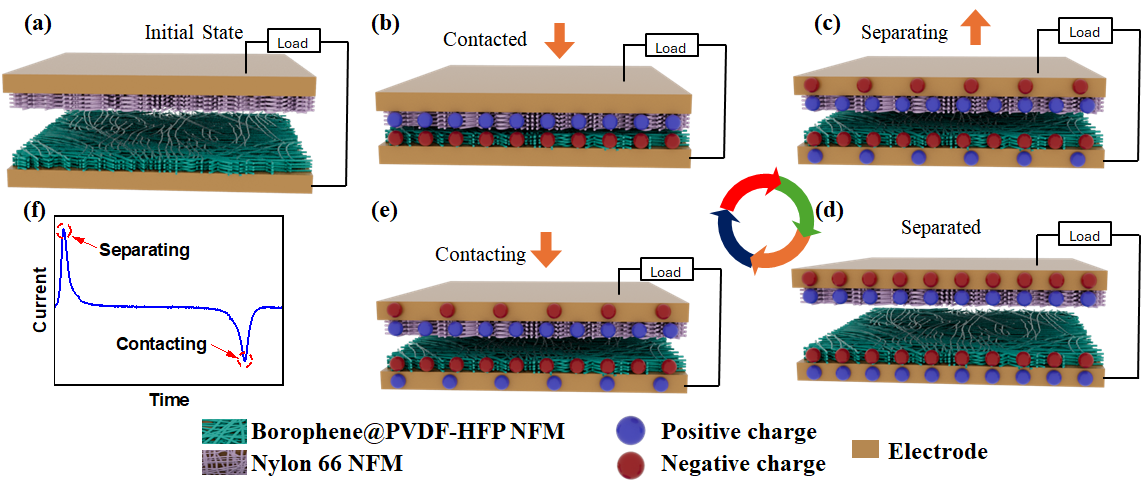


**Figure S15.** Working principle of TENG (a) Initial state (before any force applied, the two layers are fully separated). (b) Both layers came into contact. (c) Both layers start separating. (d) Both layers are fully separated. (e) Both layers are coming into contact again. (f) Short circuit current waveform.


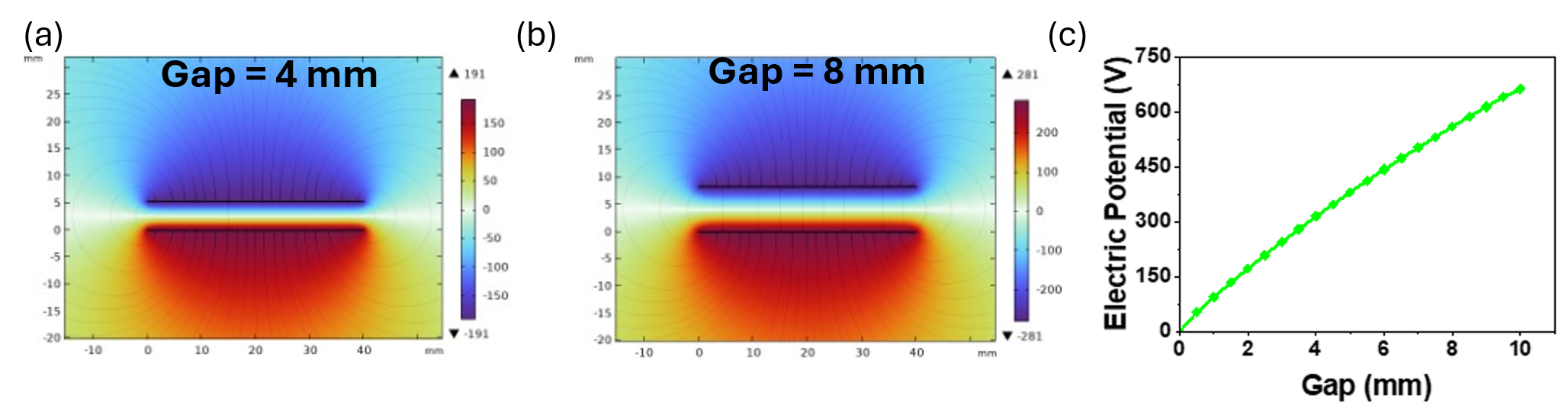


**Figure S16.** Electrical potential distribution results from the COMSOL simulation for (a) 4mm gap and (b) 8 mm gap between two dielectrics. (c) Changes of electric potential with gap.


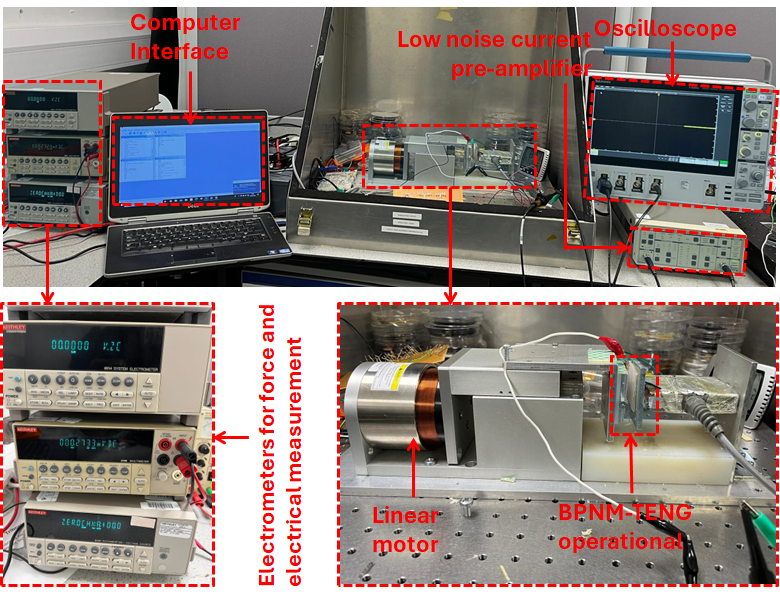


**Figure S17.** Photograph of experimental setup of the proposed CNF-TENG.

**Figure S18**. Changes of output (a) Charge density and (b) Current density with different concentrations of borophene filler into PVDF-HFP.

**Figure S19.** (a) Power density of the proposed borophene@PVDF-HFP based CNF-TENG. (b) Output voltage waveform at 30 MΩ optimal load resistance.

**Figure S20.** (a) Photographs of the humidity test setup for different humidity conditions. (b) The durability test of the proposed borophene@PVDF-HFP based CNF-TENG for 50% relative humidity.


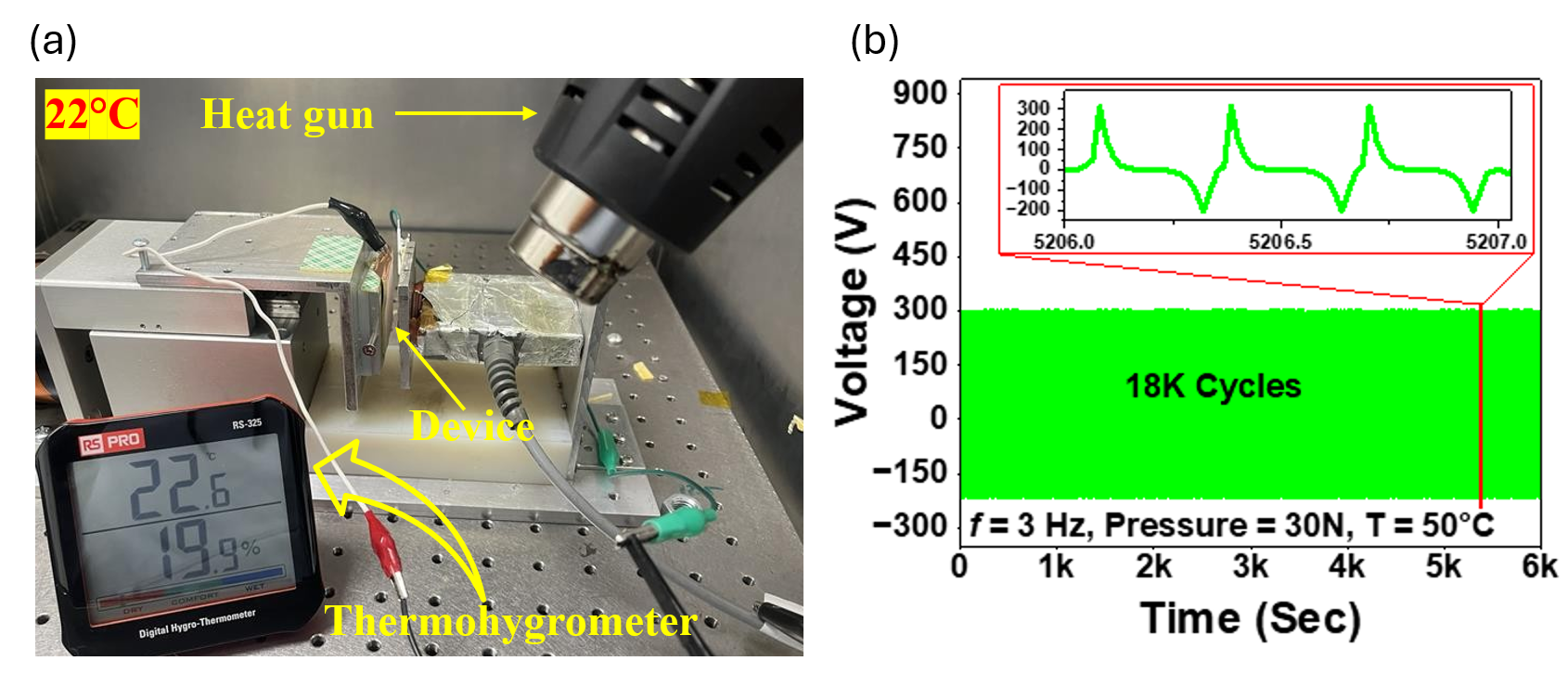


**Figure S21.** (a) Photographs of the temperature test setup for different temperature conditions. (b) The durability test of the proposed borophene@PVDF-HFP based CNF-TENG for 50°C temperature.

**Figure S22.** Changes in output voltage with different applied pressure at a 3 Hz frequency.


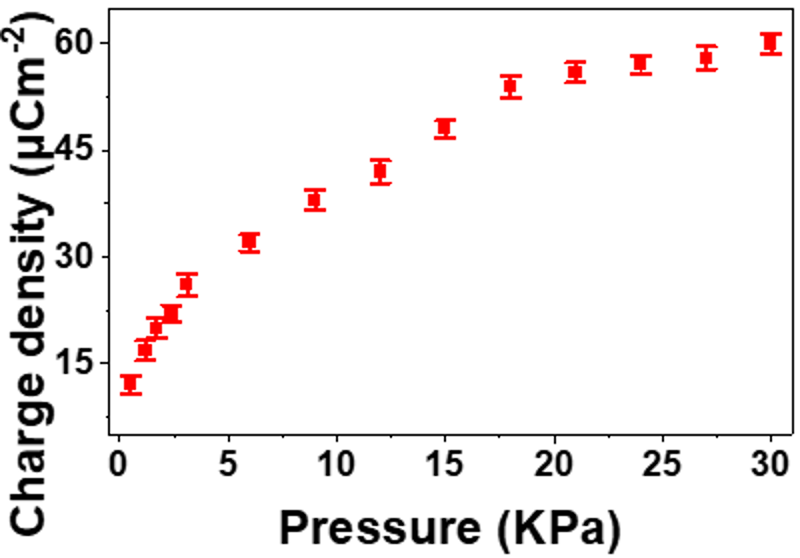


**Figure S23**. Charge density of the CNF-TENG as a function of pressure.


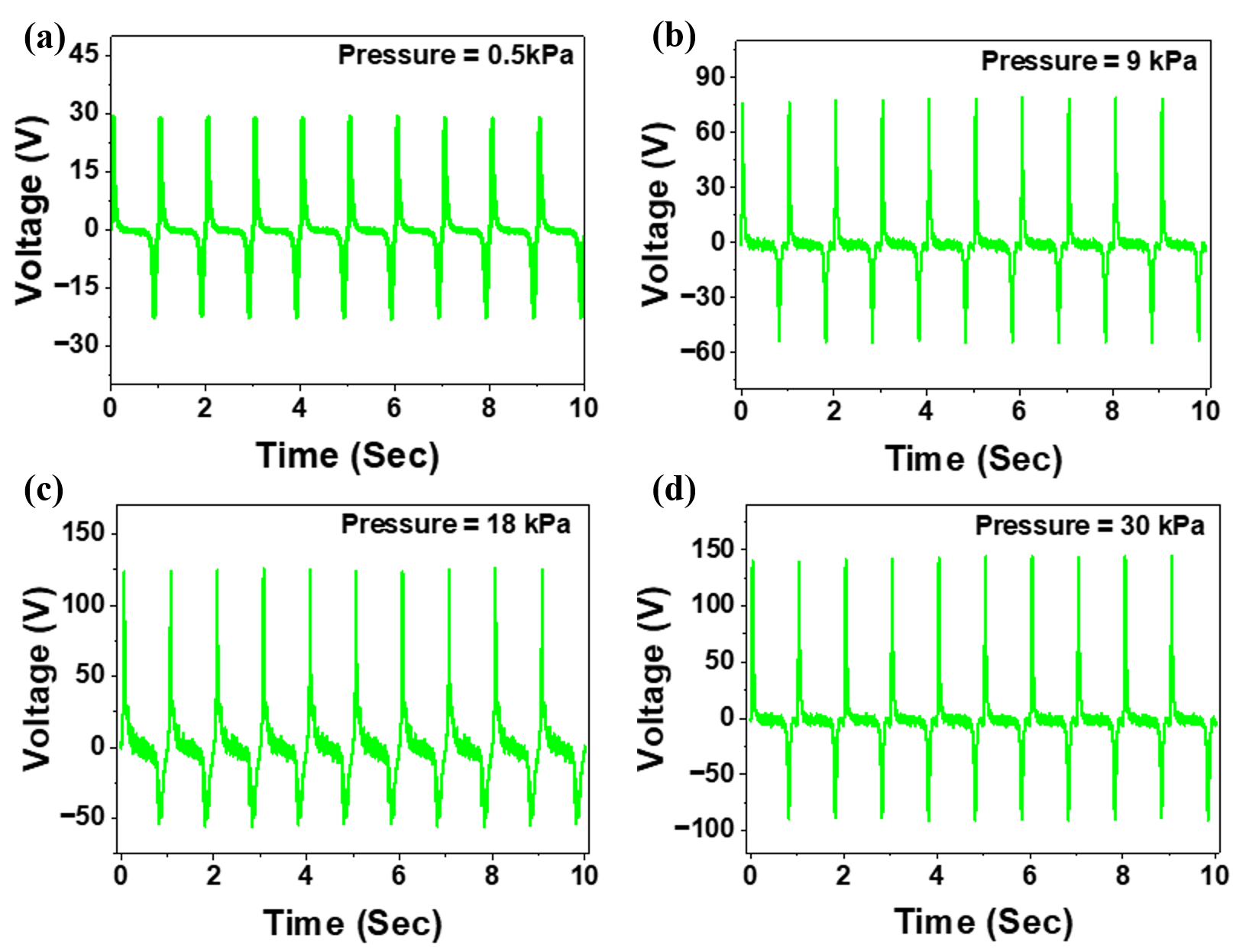


**Figure S24.** Voltage for different pressures of (a) 1 KPa (b) 9 KPa (c) 18 KPa and (d) 30 KPa at 1Hz operating frequency.

**
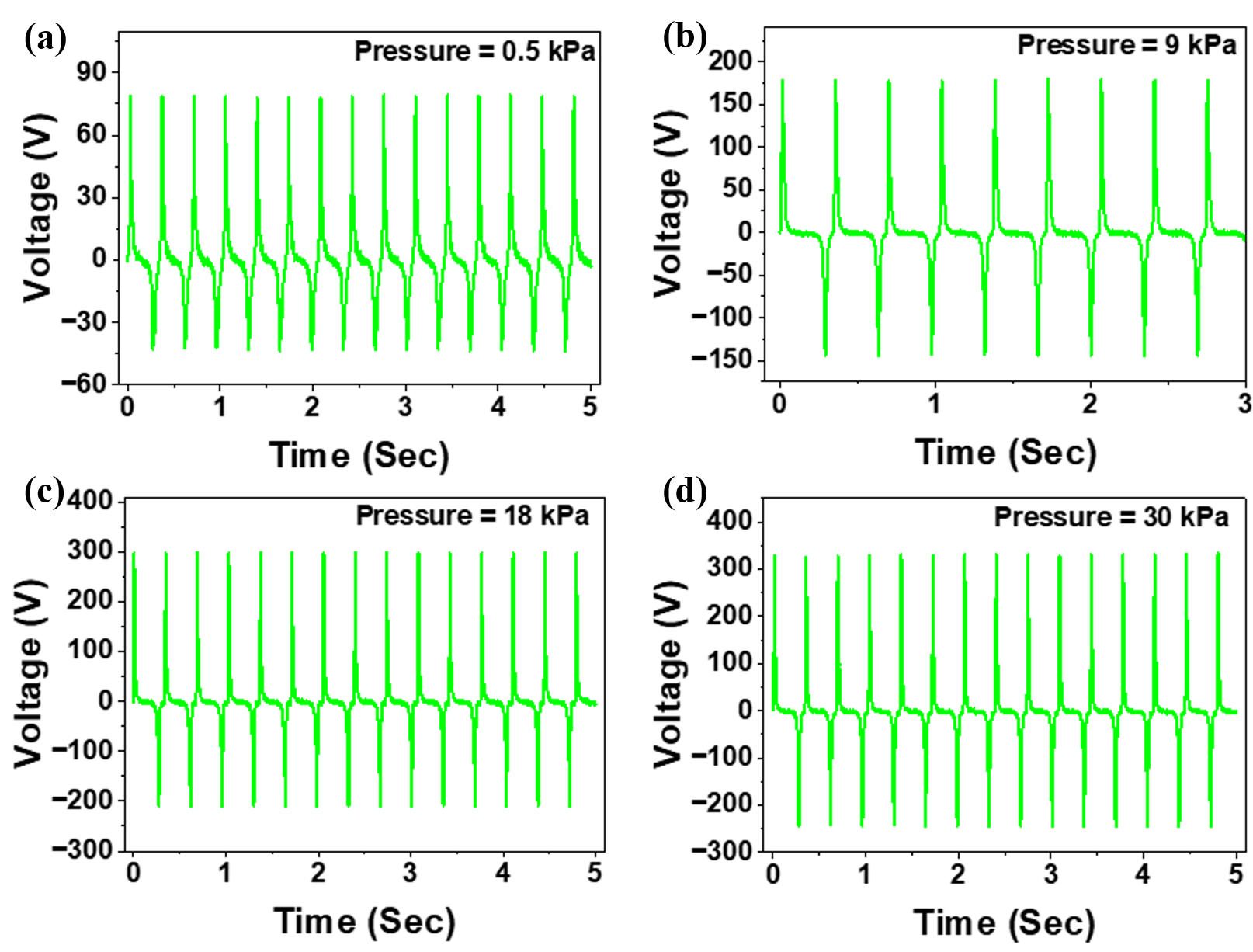
Figure S25.** Voltage for different pressures of (a) 1 KPa (b) 9 KPa (c) 18 KPa and (d) 30 KPa at 3Hz operating frequency.


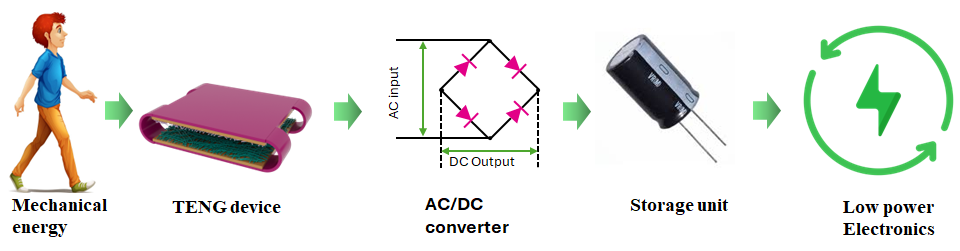


**Figure S26.** Demonstration of the borophene@PVDF-HFP based CNF-TENG as an independent energy generator for powering small, portable electronic devices by harvesting biomechanical energy from low-frequency human body-induced motion.


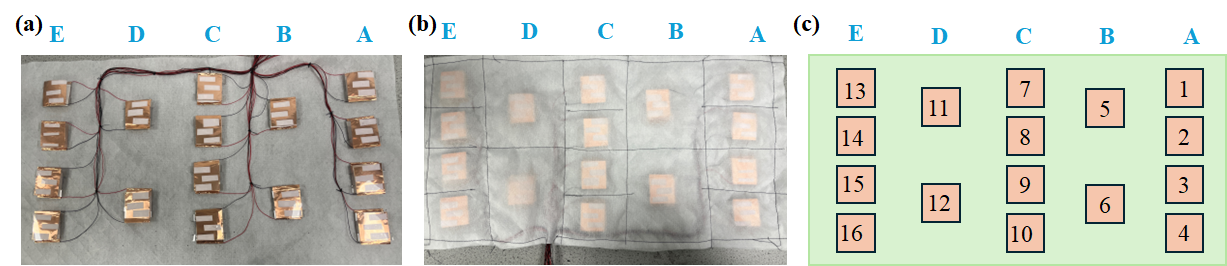


**Figure S27.** (a) Place the CNF-TENG sensor array on top of a textile and make the external wiring. (b) The sensor array is covered with the textile. (c) Position of 16 sensors divided into 5 parts from A to E.


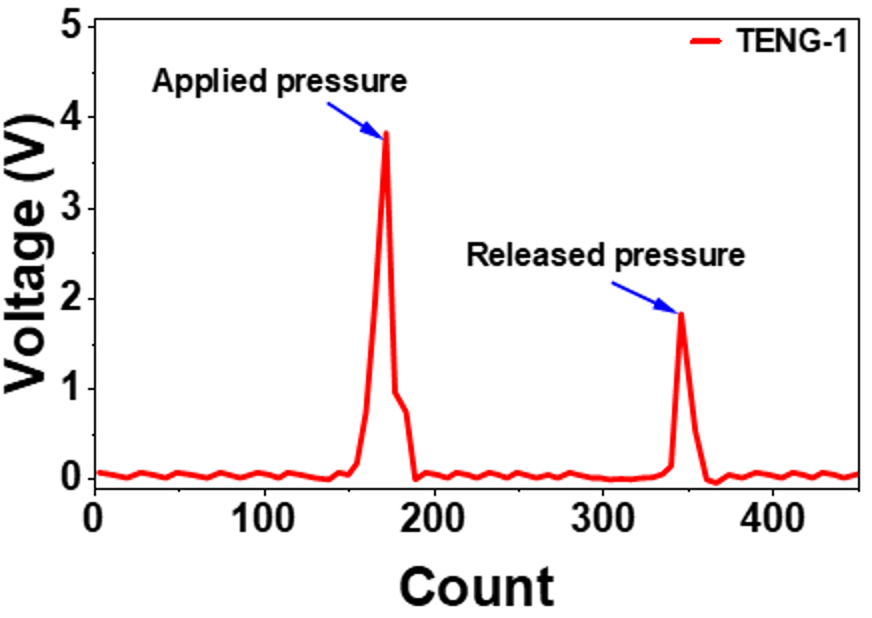


**Figure S28.** Response curve from the TENG-1 representing the applied and released signal during sleep activities.


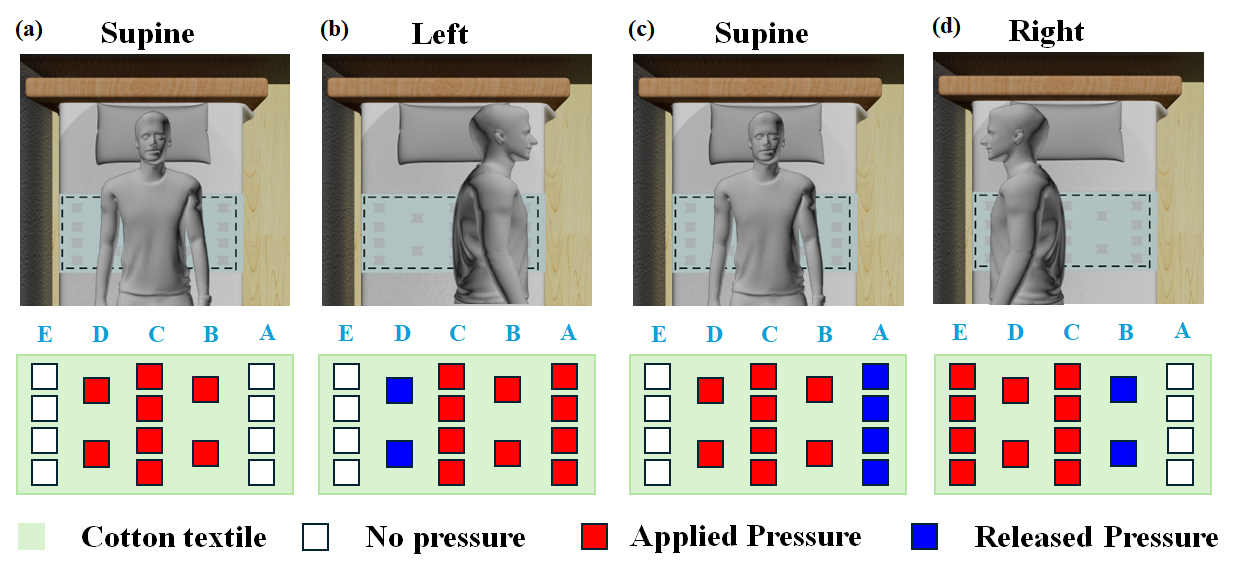


**Figure S29.** Application of the CNF-TENG sensor array as a smart bedsheet in monitoring body movement. (a) Demonstration diagrams of the body in the supine position (b) Left position, (c) Supine position, and (d) Right position.

| **Parameters** | **Value** |
| --- | --- |
| Borophene@PVDF-HFP nanofiber mat thickness | 80 µm |
| Nylon 66 nanofiber mat thickness | 100 µm |
| PET sheet | 95 µm |
| Electrode thickness | 60 µm |
| Applied force | 30 N |
| Working frequency | 3 Hz |
| Relative humidity | 30 ± 5 % |
| Temperature | Room temperature |
| Contact area of the TENG | 4 × 4 cm^2^ |
| Voltage | 510 V |
| Current | 21.3 µA |
| Charge | 120 nC |
| Power density | 1.2 Wm^-2^ |
| Load resistance | 30 MΩ |
| Sensitivity | 53.8 V kPa^-1^ |

**Table S1.** The geometry, experimental conditions and electrical features of the proposed borophene@PVDF-HFP composite TENG.

**Table S2.** Comparison of the output performance with other previously reported PVDF-HFP composite literature with this work

| **Filler** | **Process** | **Voltage (V)** | **Current (µA)** | **Charge (nC)** | **Power density (W/m2)** | **Ref.** |
| --- | --- | --- | --- | --- | --- | --- |
| SEBS | Electrospinning  electrospraying | 290 | 10 | - | 0.22 | ^[1]^ |
| Ionic liquid | Blade coating | 16.95 | 2.56 | 15.23 | .0261 | ^[2]^ |
| P3HT | Electrospinning | 78 | 7 | 42 | 0.45 | ^[3]^ |
| Sio2 | Electrospinning  electrospraying | 114.5 | 4.9 | 10.2 | 0.445 | ^[4]^ |
| MoS2 | Electrospinning | 150 | 4.2 | 59 | 0.17 | ^[5]^ |
| WS2 | Depositing | 25 | 0.14 | - | 0.54 | ^[6]^ |
| MXene | Electrospinning | 160 | 6 | 65 | 0.228 | ^[7]^ |
| Borophene | Electrospinning | 510 | 21.3 | 120 | 1.2 | This Work |

**Table S3.** Comparison of output performance with other negative tribomaterials based literatur with this work

| **Materials** | | Voltage (V) | **Power density (W/m2)** | **Stability** | Ref. |
| --- | --- | --- | --- | --- | --- |
| Positive tribo | Negative tribo |  |  |  |  |
| Graphite pencil | Paper | 106 | 0.91 | 750 | ^[8]^ |
| Nylon 66-Silk | PVDF-PET | 100 | 0.28 | 12,000 | ^[9]^ |
| Cellulose-CNT | PDMS-SnS2 | 35 | 0.13 | 10,000 | ^[10]^ |
| Nylon | PVDF-PDMS-PI | 14 | 0.05 | 10,000 | ^[11]^ |
| Nylon 6 | FEP | 200 | 0.89 | 12,000 | ^[12]^ |
| Nylon | PVDF-CsPbI3 | 180 | .023 | 1000 | ^[13]^ |
| PET | Ecoflex-BaTiO3 | 144 | 0.013 | 7200 | ^[14]^ |
| Nylon 66 | PVDF-HFP Borophene | 510 | 1.2 | 18,000 | This Work |

**Table S4.** Comparison of sensitivity with other previously reported papers

| Materials | | Pressure Range (kPa) | Frequency (Hz) | Sensitivity (V/kPa) | Year-Author | Journal Name | Ref. |
| --- | --- | --- | --- | --- | --- | --- | --- |
| Positive | Negative |  |  |  |  |  |  |
| PET/ITO | FEP | 1.25-6.25 | 3 | 46 | Zheng-2022 | Chemical Eng. J. | ^[15]^ |
| PDMS | PDMS-CNT | 5-50 | 1 | 0.51 | Rasel-2018 | Nano Energy | ^[16]^ |
| PMMA | PTFE | 0.0245-1.23 | 4 | 7.29 | Jan-2024 | Soft Science | ^[17]^ |
| Al foil | Ecoflex/Carbon Black | 0.612-8.58 | 4 | 2.57 | Jan-2024 | Soft Matter | ^[18]^ |
| Glass | Ecoflex/Ag | <5 | N/A | 9.54 | Lai-2018 | Advanced Mat. | ^[19]^ |
| Conductive Fabric | PTFE | <0.8 | 6 | 0.53 | Wu-2025 | Nano Energy | ^[20]^ |
| Nylon | PVC gel | 10-40 | 2 | 2.03 | Park-2025 | J. of Materials Chem. A | ^[21]^ |
| Nylon 66 | PVDF/Siloxene | <3 | 1 | 12 | Bhatta-2022 | AFM | ^[22]^ |
| PMMA | PDMS/Al | 0.2-2.4 | N/A | 3.11 | Ke-2020 | Small | ^[23]^ |
| TPU/Ionic Liquid | PTFE | <20 | 4 | 23.3 | Hwang-2019 | AEM | ^[24]^ |
| Cu | PDMS | 0-330 | 1.5 | 0.304 | Yi-2023 | Nano Energy | ^[25]^ |
| PET | Natural rubber/Graphene | 0.1-1.3 | N/A | 0.16 | Thapa-2024 | Small | ^[26]^ |
| Ferrofluid | PTFE | 0-2.5 | N/A | 21.48 | Liu-2022 | Nano Micro Letters | ^[27]^ |
| PEO/PolyDADMAC | PVDF-HFP | 1-25 | 4 | 21.88 | Islam-2024 | AFM | ^[28]^ |
| PEO | PVDF-HFP/V2CTx-MXene | 1-45 | 3 | 25.17 | Faruk-2024 | Nano Energy | ^[29]^ |
| Nylon 11 | PVDF-HFP/Co-NPC | 1-15 | 1 | 6.39 | Rahman-2021 | Nano Energy | ^[30]^ |
| TPU | PVDF-HFP/MXene | 0.0025-5 | 2 | 17.5 | Zhang-2024 | Nano Energy | ^[31]^ |
| SEBS | PVDF-HFP | 8-89 | 5 | 0.68 | Li-2020 | Nano Energy | ^[32]^ |
| TPU | PVDF-HFP | 0-1.2 | 5 | 0.718 | Uzabakiriho-2022 | Nano Select | ^[33]^ |
| Nylon | PVDF-HFP | 0-2.8 | 1.5 | 2.2 | Guo-2023 | Nano Energy | ^[34]^ |
| Cu | PDMS-ion get/PVDF-HFP | 0.01-1.5 | 5 | 0.43 | Lin-2018 | Nano Energy | ^[35]^ |
| Nylon 66 | PVDF-HFP/Borophene | 0.5-3.125 | 1 | 18.96±0.6 | This Work | | |
|  |  |  | 3 | 53.8±1.2 |  |  |  |
|  |  | 3.125-18 | 1 | 6.71±0.2 |  |  |  |
|  |  |  | 3 | 16.8±0.3 |  |  |  |

**References**

[1] Y. Li, J. Xiong, J. Lv, J. Chen, D. Gao, X. Zhang, P. S. Lee, *Nano Energy* **2020**, *78*, 105358.

[2] D. L. Vu, C. P. Vo, C. D. Le, K. K. Ahn, *Int J Energy Res* **2021**, *45*, 8960.

[3] M.-F. Lin, K.-W. Chang, C.-H. Lee, X.-X. Wu, Y.-C. Huang, *Sci Rep* **2022**, *12*, 14842.

[4] C. Fan, Z. Long, Y. Zhang, A. Mensah, H. He, Q. Wei, P. Lv, *Nano Energy* **2023**, *116*, 108842.

[5] B. Amrutha, J. U. K. Yoon, I. Woo, P. Gajula, A. A. Prabu, J. W. Bae, *Appl Mater Today* **2024**, *41*, 102503.

[6] M. Baraily, B. Baro, R. Boruah, S. Bayan, *Nanotechnology* **2024**, *35*, 365502.

[7] Y. Zhang, X. Dai, Y. Zhou, J. Shao, L. Zhao, T. Wang, F. Liu, X. Yan, M. Yang, P. Sun, G. Lu, *Nano Energy* **2024**, *132*, 110372.

[8] G. Ferreira, A. Opinião, S. Das, S. Goswami, L. Pereira, S. Nandy, R. Martins, E. Fortunato, *Nano Energy* **2022**, *95*, 107021.

[9] S. Bairagi, G. Khandelwal, X. Karagiorgis, S. Gokhool, C. Kumar, G. Min, D. M. Mulvihill, *ACS Appl Mater Interfaces* **2022**, *14*, 44591.

[10] K.-Y. Hsu, S.-M. Huang, B. T. Murti, C.-C. Chen, Y.-C. Chao, I.-C. Ha, C.-C. Tsai, C.-Y. Chen, M.-L. Tsai, P.-K. Yang, *Nano Energy* **2025**, *134*, 110501.

[11] W. Akram, Q. Chen, X. Zhang, S. Ren, L. Niu, J. Fang, *Nano Energy* **2024**, *131*, 110275.

[12] E. J. Jelmy, M. Sunil, C. Kandappanthodi, P. Rincy, K. J. Saji, S. C. Pillai, H. John, *Journal of Physics: Energy* **2024**, *6*, 025010.

[13] S. Mondal, S. Maiti, T. Paul, S. Poddar, B. K. Das, K. K. Chattopadhyay, *ACS Appl Mater Interfaces* **2024**, *16*, 9231.

[14] W.-T. Guo, Y. Lei, X.-H. Zhao, R. Li, Q.-T. Lai, S.-Z. Liu, H. Chen, J.-C. Fan, Y. Xu, X.-G. Tang, Q.-J. Sun, Q. Sun, *Nano Energy* **2024**, *131*, 110324.

[15] Z. Zheng, D. Yu, B. Wang, Y. Guo, *Chemical Engineering Journal* **2022**, *446*, DOI 10.1016/j.cej.2022.137393.

[16] M. S. Rasel, P. Maharjan, M. Salauddin, M. T. Rahman, H. O. Cho, J. W. Kim, J. Y. Park, *Nano Energy* **2018**, *49*, 603.

[17] A. A. Jan, S. Kim, S. Kim, *Soft Science* **2024**, *4*, DOI 10.20517/ss.2023.54.

[18] A. Aamir Jan, S. Kim, S. Kim, *Soft Matter* **2024**, *20*, 6558.

[19] Y. C. Lai, J. Deng, R. Liu, Y. C. Hsiao, S. L. Zhang, W. Peng, H. M. Wu, X. Wang, Z. L. Wang, *Advanced Materials* **2018**, *30*, DOI 10.1002/adma.201801114.

[20] Y. Wu, Y. Li, X. Wang, P. Guo, H. Luo, X. Li, X. Zhu, J. Yang, *Nano Energy* **2025**, *134*, DOI 10.1016/j.nanoen.2024.110581.

[21] H. Park, Y. Ryu, H. Joo, S. Gwak, G. S. Gbadam, S. Niu, J. H. Lee, *J Mater Chem A Mater* **2025**, *13*, 4197.

[22] T. Bhatta, S. Sharma, K. Shrestha, Y. Shin, S. Seonu, S. Lee, D. Kim, M. Sharifuzzaman, S. S. Rana, J. Y. Park, *Adv Funct Mater* **2022**, *32*, DOI 10.1002/adfm.202202145.

[23] K. H. Ke, C. K. Chung, *Small* **2020**, *16*, DOI 10.1002/smll.202001209.

[24] H. J. Hwang, J. S. Kim, W. Kim, H. Park, D. Bhatia, E. Jee, Y. S. Chung, D. H. Kim, D. Choi, *Adv Energy Mater* **2019**, *9*, 1803786.

[25] P. Yi, X. Fu, Y. Liu, X. Zhang, C. Zhang, X. Li, *Nano Energy* **2023**, *113*, DOI 10.1016/j.nanoen.2023.108592.

[26] S. Sharma, A. Thapa, S. Pramanik, C. Sengupta, T. Mondal, *Small* **2024**, *20*, DOI 10.1002/smll.202404771.

[27] J. Liu, Z. Wen, H. Lei, Z. Gao, X. Sun, *Nanomicro Lett* **2022**, *14*, DOI 10.1007/s40820-022-00831-7.

[28] M. Robiul Islam, O. Faruk, S. M. S. Rana, G. B. Pradhan, H. Kim, M. S. Reza, T. Bhatta, J. Y. Park, *Adv Funct Mater* **2024**, *34*, DOI 10.1002/adfm.202403899.

[29] O. Faruk, M. R. Islam, S. M. S. Rana, G. B. Pradhan, H. S. Kim, M. Asaduzzaman, T. Bhatta, J. Y. Park, *Nano Energy* **2024**, *127*, DOI 10.1016/j.nanoen.2024.109787.

[30] M. T. Rahman, S. S. Rana, M. A. Zahed, S. Lee, E. S. Yoon, J. Y. Park, *Nano Energy* **2022**, *94*, DOI 10.1016/j.nanoen.2022.106921.

[31] Y. Zhang, X. Dai, Y. Zhou, J. Shao, L. Zhao, T. Wang, F. Liu, X. Yan, M. Yang, P. Sun, G. Lu, *Nano Energy* **2024**, *132*, DOI 10.1016/j.nanoen.2024.110372.

[32] Y. Li, J. Xiong, J. Lv, J. Chen, D. Gao, X. Zhang, P. S. Lee, *Nano Energy* **2020**, *78*, DOI 10.1016/j.nanoen.2020.105358.

[33] P. C. Uzabakiriho, M. Wang, C. Ma, G. Zhao, *Nanoscale* **2022**, *14*, 6600.

[34] W. Guo, Y. Xia, Y. Zhu, S. Han, Q. Li, X. Wang, *Nano Energy* **2023**, *108*, 108229.

[35] M. F. Lin, J. Xiong, J. Wang, K. Parida, P. S. Lee, *Nano Energy* **2018**, *44*, 248.
